# Development of a patient-specific model of the human coronary system for percutaneous transluminal coronary angioplasty balloon catheter training and testing

**DOI:** 10.1186/s12938-024-01271-7

**Published:** 2024-08-30

**Authors:** C. Amstutz, M. Ilic, N. Fontaine, L. Siegenthaler, J. Illi, A. Haeberlin, A. Zurbuchen, J. Burger

**Affiliations:** 1 School of Biomedical and Precision Engineering, Faculty of Medicine, University of Bern, Güterstrasse 24/26, CH-3010 Bern, Switzerland; 2 Department of Cardiology, Inselspital, Bern University Hospital, University of Bern, Bern, Switzerland

**Keywords:** Patient-specific phantoms, 3DPSP, Silicone compliance, Friction, Additive manufacturing, PTCA balloon catheter

## Abstract

**Background:**

To treat stenosed coronary arteries, percutaneous transluminal coronary angioplasty (PTCA) balloon catheters must combine pushability, trackability, crossability, and rewrap behavior. The existing anatomic track model (ASTM F2394) for catheter testing lacks 3D morphology, vessel tortuosity, and compliance, making evaluating performance characteristics difficult. This study aimed to develop a three-dimensional patient-specific phantom (3DPSP) for device testing and safe training for interventional cardiologists.

**Methods:**

A range of silicone materials with different shore hardnesses (00–30–45 A) and wall thicknesses (0.5 mm, 1 mm, 2 mm) were tested to determine compliance for creating coronary vessel phantoms. Compliance was assessed using optical coherence tomography (OCT) and compared to values in the literature. Stenosis was induced using multilayer casting and brushing methods, with gypsum added for calcification. The radial tensile properties of the samples were investigated, and the relationship between Young’s modulus and compliance was determined. Various methods have been introduced to approximate the friction between silicone and real coronary vessel walls. Computerized tomography (CT) scans were used to obtain patient-specific anatomy from the femoral artery to the coronary arteries. Artery lumens were segmented from the CT scans to create dissolvable 3D-printed core models.

**Results:**

A 15A shore hardness silicone yielded an experimental compliance of 12.3–22.4 $\frac{m{m}^{2}}{mmHg}\cdot {10}^{3}$ for stenosed tubes and 14.7–57.9 $\frac{m{m}^{2}}{mmHg}\cdot {10}^{3}$ for uniform tubes, aligning closely with the literature data (6.28–40.88 $\frac{m{m}^{2}}{mmHg}\cdot {10}^{3}$). The Young’s modulus ranged from 43.2 to 75.5 kPa and 56.6–67.9 kPa for the uniform and calcified materials, respectively. The dependency of the compliance on the wall thickness, Young’s modulus, and inner diameter could be shown. Introducing a lubricant reduced the silicone friction coefficient from 0.52 to 0.13. The 3DPSP was successfully fabricated, and comparative analyses were conducted among eight commercially available catheters.

**Conclusion:**

This study presents a novel method for crafting 3DPSPs with realistic mechanical and frictional properties. The proposed approach enables the creation of comprehensive and anatomically precise setups spanning the right femoral artery to the coronary arteries, highlighting the importance of such realistic environments for advancing medical device development and fostering safe training conditions.

**Supplementary Information:**

The online version contains supplementary material available at 10.1186/s12938-024-01271-7.

## Background

Newly developed medical devices, such as percutaneous transluminal coronary angiography (PTCA) balloon catheters, can be assessed during the preclinical phase using human cadavers [1, 2] or animal models [3]. The pig model is frequently employed to study atherosclerosis in larger animals. Like in other models, atherosclerosis is induced in pigs through a high-fat diet. A notable benefit of the pig model is its size, which allows imaging tools to examine plaque growth and readily available clinical devices for analysis. [4, 5]

Using animals for device testing invariably raises ethical concerns, especially when disease induction is necessary. Moreover, such trials entail high costs and pose challenges when comparing devices due to inherent variations among animals. Therefore, manufacturers often also rely on results from simulated use-case tests to evaluate the performance of such devices. One example is the test on an anatomic track model (ASTM F2394). These models provide reproducible results and provide insights into the performance of catheters. However, they do not represent the human cardiovascular system's 3-dimensional (3D) anatomy. Furthermore, vessel tortuosity is known to impact percutaneous coronary intervention (PCI) delivery [6, 7]. Due to the 2D structure and the design of the ASTM model, tortuosity is present only to a limited extent.

A systematic review by Bernhard et al. [8] showed that 3D patient-specific phantoms (3DPSP) are already recognized in cardiology for preoperative planning to aid decision-making and improve interventions. However, their use is not widespread, and further evidence is needed. Ormiston et al. [9] highlighted the importance of bench testing for coronary bifurcation treatment on realistic models considering the anatomy, flow conditions, and vascular compliance to assess stenting techniques, stents, or scaffold testing. They further presented some guidelines regarding the anatomy of bifurcation models, including scaling laws for flow and testing. In [10–12], 3DPSP models of the coronary arteries are shown and used for preoperative planning in complex cases or anomalous anatomy. However, neither of these studies considered the mechanical properties of the coronary arteries, and Watanabe et al. [10] and Gach et al. [12] considered only the aortic and coronary anatomy.

The biological arterial wall consists of multiple layers. Therefore, Brunette et al. [13] manufactured a coronary artery phantom for particle image velocimetry featuring multiple layers. The multilayered phantom is based on translucent silastic T-2 silicone and is produced with five molds. An asymmetric narrowing simulated 50% occlusion. Brunette et al.'s approach to creating anatomically more realistic phantoms was suitable for blood flow investigations. However, the study did not account for the anatomical structure of the coronary arteries or the realistic shapes of plaques, and evidence of realistic mechanical properties was lacking. Yazdi et al. [14] provided a comprehensive review of models for particle image velocimetry, suggesting the use of a female mold to delineate the outer surface of silicone while accurately positioning a male mold within it to define the inner surface of compliant phantoms.

Finn et al. [15] manufactured flexible coronary arteries with compliance of $21- 37 \frac{m{m}^{2}}{mmHg}\cdot {10}^{3}$ using the lost wax technique. However, the proposed method requires several time-consuming steps (manufacturing two metallic molds, the lost core, and the arterial wall, six freeze‒thaw cycles with a total duration of 72 h, and removal of the core) to generate the final geometry, and only the aortic and coronary anatomy was considered. In Biglino et al. [16], PolyJet 3D printing technology was used to manufacture phantoms using a layer-by-layer (16 µm) approach. Phantoms of the descending aorta were produced at 50 mm in length and 15.5 mm in inner diameter using the material TangoPlus FullCure^®^.

This study aimed to introduce a modular 3DPSP that mimics the anatomical structure from the femoral artery to the coronary arteries. The developed model incorporates the coronary arteries' realistic mechanical and frictional properties and is designed for PTCA balloon catheter testing. Using a realistic model, PTCA balloon catheters can be evaluated in real-case scenarios. This knowledge can contribute to understanding the behavior of balloon catheters, advancing their development, and increasing their performance.

Additionally, this phantom facilitates realistic simulation of PTCA procedures and serves as an educational resource for healthcare professionals seeking to enhance their procedural skills and gain hands-on experience in a safe and controlled environment before performing interventions on patients. By offering physicians a tangible representation of coronary anatomies, this model aids in preprocedural planning, allowing for the development of more tailored treatment strategies and ultimately contributing to improved patient outcomes.

## Results

From the material screening, it was found that compliance can be adjusted by either introducing a different material or changing the WT. However, small WTs are considered unfavorable since they are easily damaged. Therefore, the ELASTOSIL^®^ VARIO 15 was used for further testing.

### Compliance

After the material screening, further tests were performed on uniform cast tubes with an inner diameter of 3 mm made from ELASTOSIL^®^ VARIO 15 (*n* = 4), and compliance was measured. The results are shown in Table 1. Compliance declines when the WT increases. Furthermore, small WTs and large tubes show an increase in scattering.

**Table 1:** Compliance values for the uniform silicone tubes made by ELASTOSIL^®^ VARIO 15 presented as the median (IQR) and the corresponding Pearson’s linear correlation coefficient $r$ and the cross-sectional area at a pressure of 120 mmHg

Uniform WT $\left[mm\right]$	MLA $\left[m{m}^{2}\right]$	${A}_{120}\left[m{m}^{2}\right]$	$CC \left[\frac{{mm}^{2}}{mmHg}\cdot {10}^{3}\right]$	$r \left[-\right]$
0.5	5.9 (0.6)	10.5 (0.6)	57.9 (10.5)	0.991 (0.009)
0.75		8.8 (0.5)	30.7 (1.5)	0.999 (0.001)
1		8.9 (0.02)	31.0 (4.8)	0.993 (0.002)
2		7.7 (0.3)	14.7 (5.1)	0.989 (0.026)
0.75	16.43 (2.5)	31.1 (2)	243 (156.2)	0.983 (0.011)
1		35.2 (6)	182 (55.1)	0.986 (0.007)

**Fig. 1:**
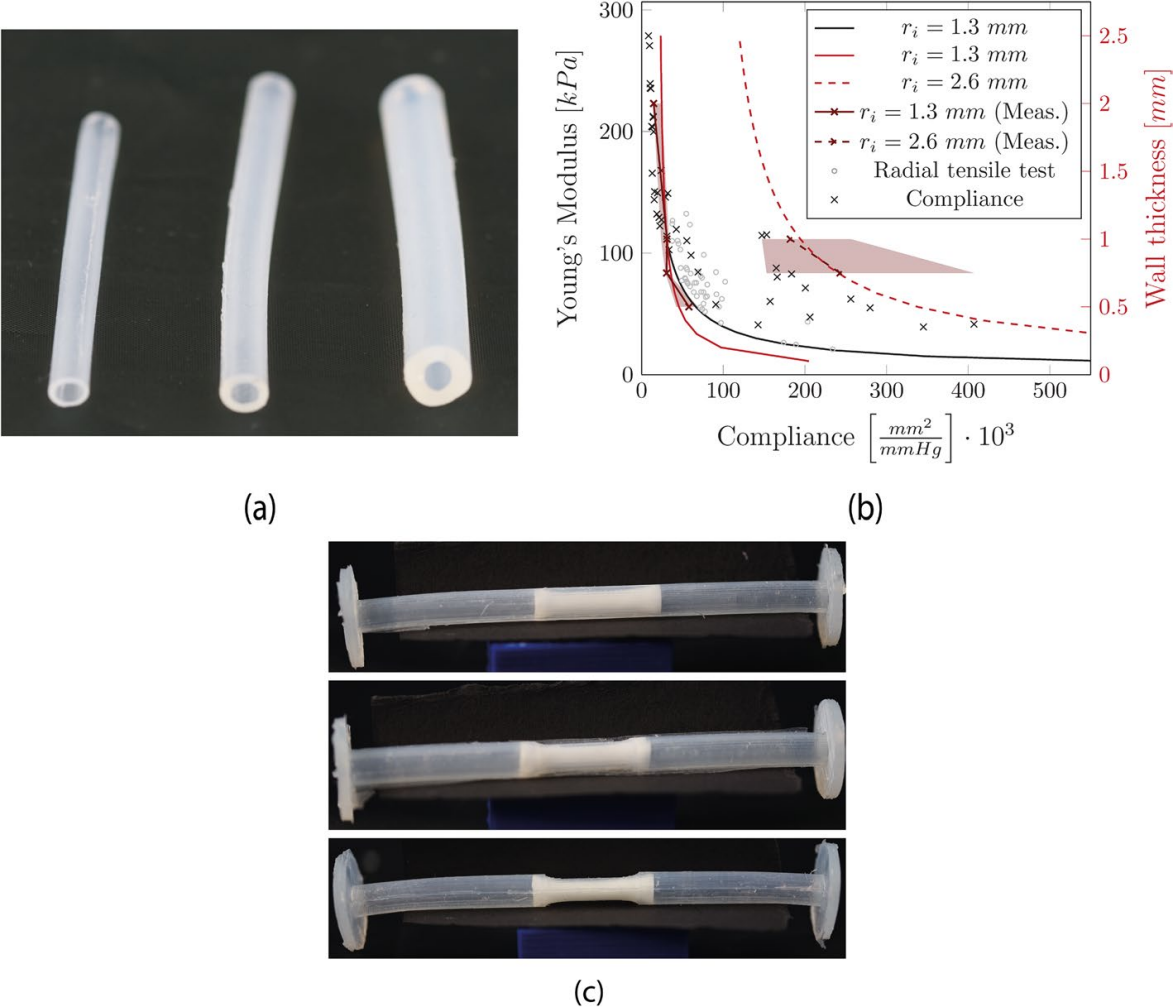
**a** Uniform silicone tubes with WTs of 0.5 mm, 1 mm, and 2 mm (from left to right). **b** Compliance is based on Young’s modulus (black) and the WT (red). Gray: converted compliance from the radial tensile tests. Green: converted Young’s modulus from the compliance test on uniform silicone tubes; yellow: converted Young’s modulus from the compliance test on stenosed silicone tubes. **c** Typical stenosed silicone tubes. Top: thick silicone tube with calcification; middle: medium silicone tube with calcification; bottom: medium stenosed silicone tube without calcification

Casted tubes with WTs of 0.5 m, 1 mm, and 2 mm are shown in Fig. 1a. Compliance was calculated based on Eqs. 2, 4. The result is plotted in Fig. 1b. For the dependency of the compliance on Young’s modulus, a tube with a ${r}_{i}=1.3$ mm, and a WT of 1 mm is assumed (black curve). As expected, an increase in Young’s modulus causes a reduction in compliance. Furthermore, an increase in the WT results in a reduction in compliance (red curve). Additionally, a large ${r}_{i}$ shows a higher compliance (red dashed line). These curves were calculated using Young’s modulus of 110 kPa. The same becomes visible from the results of the measurement of compliance, which depends on the WT shown in Table 1. However, it can also be seen that the measurement of large inner dimensions shows increased scattering (shaded area). Overall, the median values of the measurements agree with the calculated compliance values. Furthermore, the experimentally obtained compliance was converted to Young’s modulus (Eq. 3) and vice versa (Eq. 4). The compliance vs. Young’s modulus is shown as gray marks for the results of the radial tensile test and black marks for the compliance measurement.

Furthermore, stenotic tubes were produced by using the casting method. The WT at the stenosis was 1.07 (0.09) for thin silicone tubes, 1.46 (0.19) for medium silicone tubes, and 2.03 (0.07) for thick silicone tubes (Fig. 1 c).

The results of the compliance measurements are shown in Table 2. For tubes without calcification, only one sample could be measured.

**Table 2:** Results of the compliance measurement and the corresponding Pearson’s linear correlation coefficient $r$ and the cross-sectional area at a pressure of 120 mmHg on stenosed tubes with calcification

Sample	Calcified layer thickness [%]	MLA $\left[m{m}^{2}\right]$	${A}_{120} \left[m{m}^{2}\right]$	$CC \left[\frac{{mm}^{2}}{mmHg}\cdot {10}^{3}\right]$	$r \left[-\right]$
Thick	53 (19)	4.84 (0.3)	6.1 (0.5)	12.3 (7.7)	0.97 (0.015)
Medium	49 (11)		5.8 (0.3)	10.5 (0.6)	0.994 (0.01)
Thin	39 (4)		6.4 (0.5)	16.5 (8.9)	0.99 (0.018)
Thick	-	4.5 (0.1)	5.8 (0.4)	14.27 (2.4)	0.99 (0.008)
Medium	-		5.8	15.9	0.968
Thin	-		6.81	22.4	0.991

### Radial tensile properties

**Fig. 2:**
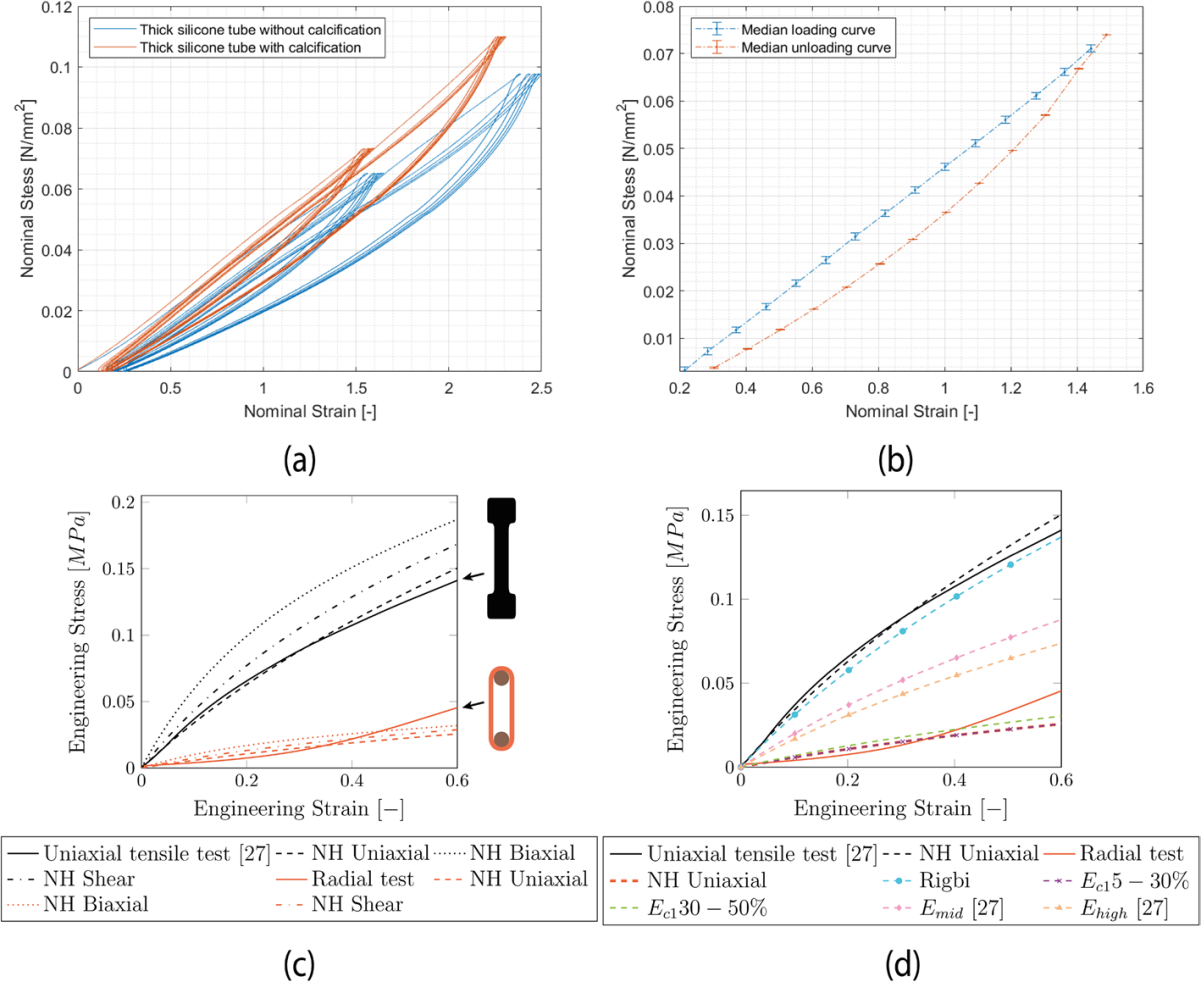
**a** Resulting nominal stress‒strain curve for a thick stenosed silicone tube with and without calcification. **b** Median loading and unloading curve for loading to 5 N. **c** Results of the neo-Hookean model with its multiaxial behavior (dashed lines, uniaxial, biaxial, shear) based on the input of uniaxial tensile test data (black) from [26] and the radial tensile test (red). **d** The result of the neo-Hookean model for uniaxial loading (dashed lines) is based on a different Young´s modulus compared to the measurements

During the tensile tests, hysteresis was present in all specimens; see Fig. 2a. The median curve of cycle two until cycle five is shown in Fig. 2b. Elastomers commonly exhibit preconditioning behavior, where the cycles gradually shift toward a larger strain and eventually stabilize. Therefore, it is important to note that the first cycle was analyzed separately due to its significant difference from subsequent cycles.

Young's modulus was evaluated within the 30–50% range and in a high strain region between 150 and 200% for the median cycle 5 to 6 curve. The first cycle was evaluated separately between 5 and 30% and 30–50%. These tests showed no significant difference between the brushed and casted specimens (*p* > 0.5). Furthermore, the difference between the stenosed tubes with calcification and those with pure silicone was significant only for the low-strain region of the first cycle (*p* < 0.001). The Young’s moduli can be found in Table 3. The individual values are shown in the supplementary material. High scattering was observed for all the samples.

**Table 3:** Values of the Young’s modulus measured in the radial direction on uniform and stenosed tubes presented as the median (IQR)

Specimen	Range %	${E}_{C1} \left[kPa\right]$	${E}_{loading} \left[kPa\right]$	${E}_{unloading} \left[kPa\right]$
Uniform	5–30	43.2 (14.6)	–	–
	30–50	75.5 (18.4)	70.5 (21.1)	39 (17.7)
	150–200	–	67.3 (21.2)	82.3 (26.6)
Stenosed	5–30	56.6 (20.9)	–	–
	30–50	67.9 (28.1)	62.6 (20.4)	36.1 (7.4)
	150–200	–	62.7 (18.2)	72.3 (30.6)

Figure 2c presents the results of the neo-Hookean model obtained through curve fitting to the experimental data. The model shows good agreement with the results from the uniaxial tensile test. Additionally, the dependency of the hyperelastic model on different deformation modes is demonstrated for both biaxial and shear states. The behavior of the radial tensile test can only be described in the low-strain region. The plot shows the difference between the uniaxial and radial tensile tests. Figure 2d shows good agreement between the uniaxial tensile test and the model based on Young´s modulus from Eq. 11. The Young’s moduli obtained in [26] for the low-strain region (0–20%) are similar to those obtained with Eq. 8. Therefore, this curve is not shown. The Young`s moduli at higher strain regions (20–40% and 40–60%) underestimate the behavior. The Young´s moduli from the radial tensile test resulted in a similar behavior as in Fig. 2c.

The material parameters required for the neo-Hookean hyperelastic material constitutive model (Eq. 8) based on the Young´s modulus converted from the shore hardness, the results of the radial tensile test, and the uniaxial tensile test performed in [26] are shown in Table 4.

**Table 4:** Material parameters for the neo-Hookean model based on Young’s modulus and curve fitting to the experimental data

Input	Young’s modulus [kPa]	Shear modulus [kPa]	C10 [kPa]
Equation 11	340.1	113.4	56.7
${E}_{C1}$ 5–30%	43.0	14.4	7.2
${E}_{C1}$ 30–50%	75.5	25.2	12.6
${E}_{low }$[26]	346.0	115.3	57.7
${E}_{mid }$[26]	218.0	72.7	36.3
${E}_{high}$[26]	183.0	61.0	30.5
Input curve (radial tensile tests)	–	–	10.6
Input curve (uniaxial tensile tests [26])	–	–	62.2

### Frictional properties

**Fig. 3:**
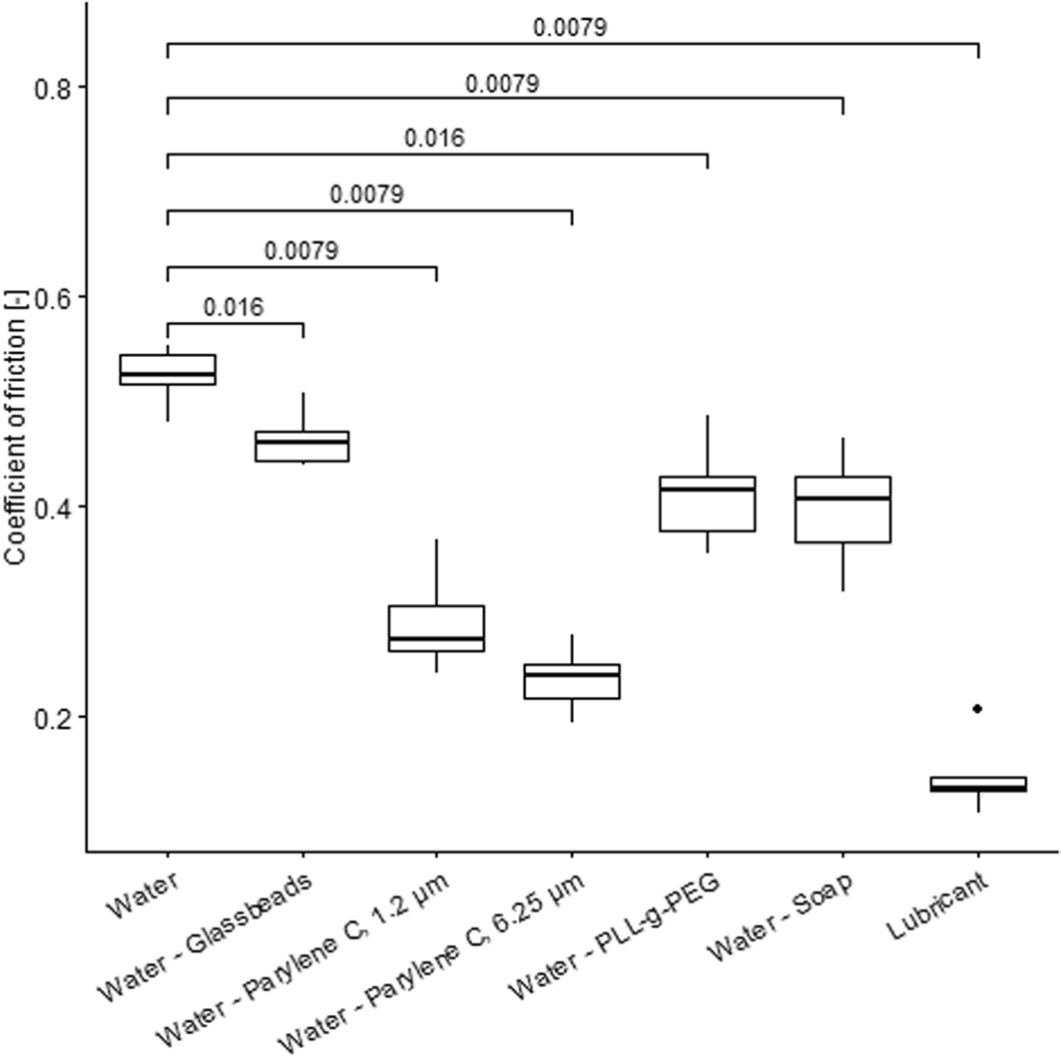
The coefficient of friction measurements, including the p value based on the Wilcoxon signed-rank test, were compared to those of plain silicone in water

The results of the friction measurements can be found in Fig. 3. The friction coefficients of the applied methods are significantly lower than that of the reference measurement with plain water [0.52 (0.03)]. The lowest friction was measured using the lubricant [0.13 (0.014)]. The PLL-g-PEG [0.42 (0.05)] and the water–soap mixture [0.41 (0.06)] were comparable.

The significance level was corrected using the Bonferroni–Holm correction $\left(\frac{p}{n}\right)$ to avoid false positive results when performing multiple tests (*n* = 6). Therefore, a *p* value of <  = 0.008 was considered significant for this investigation.

**Fig. 4:**
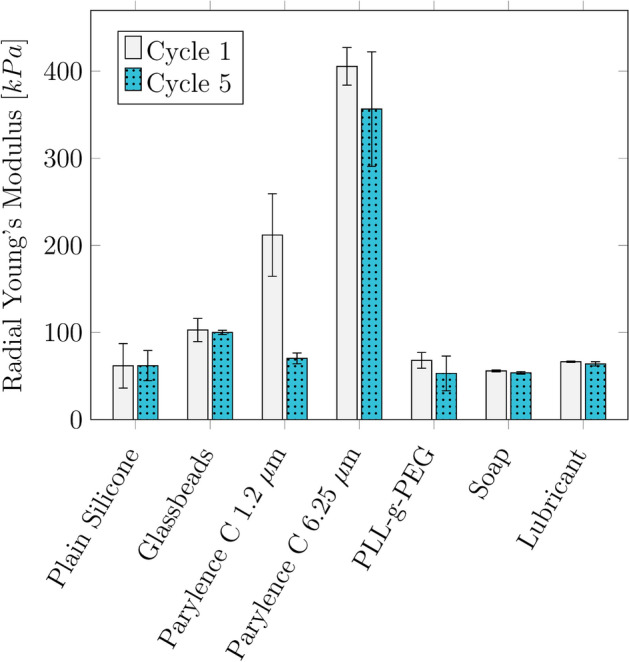
Median radial Young’s modulus of the coated samples for cycle 1 and cycle 5

The manufactured samples had a median WT of 1.39 (0.26) mm and a median length of 15.2 (0.79) mm. The influence of the coating on the Young's modulus, evaluated in the strain region of 10–30%, is shown in Fig. 4. The plain silicone had a Young's modulus of 61.8 (27.9) kPa. The silicone with embedded glass beads had a Young’s modulus of 102.7 (32.5) kPa. For Parylene C with a WT of 1.2 µm, the first cycle showed a 67% greater Young's modulus of 211.9 (94) kPa than did the later cycles with 70.3 (10.4) kPa. The thick Parylene C had the highest Young's modulus of 405.5 (93.6) kPa. The Young’s modulus decreased by 12% in the later cycles.

### 3DPSP and comparison of commercial PTCA balloon catheters

**Fig. 5:**
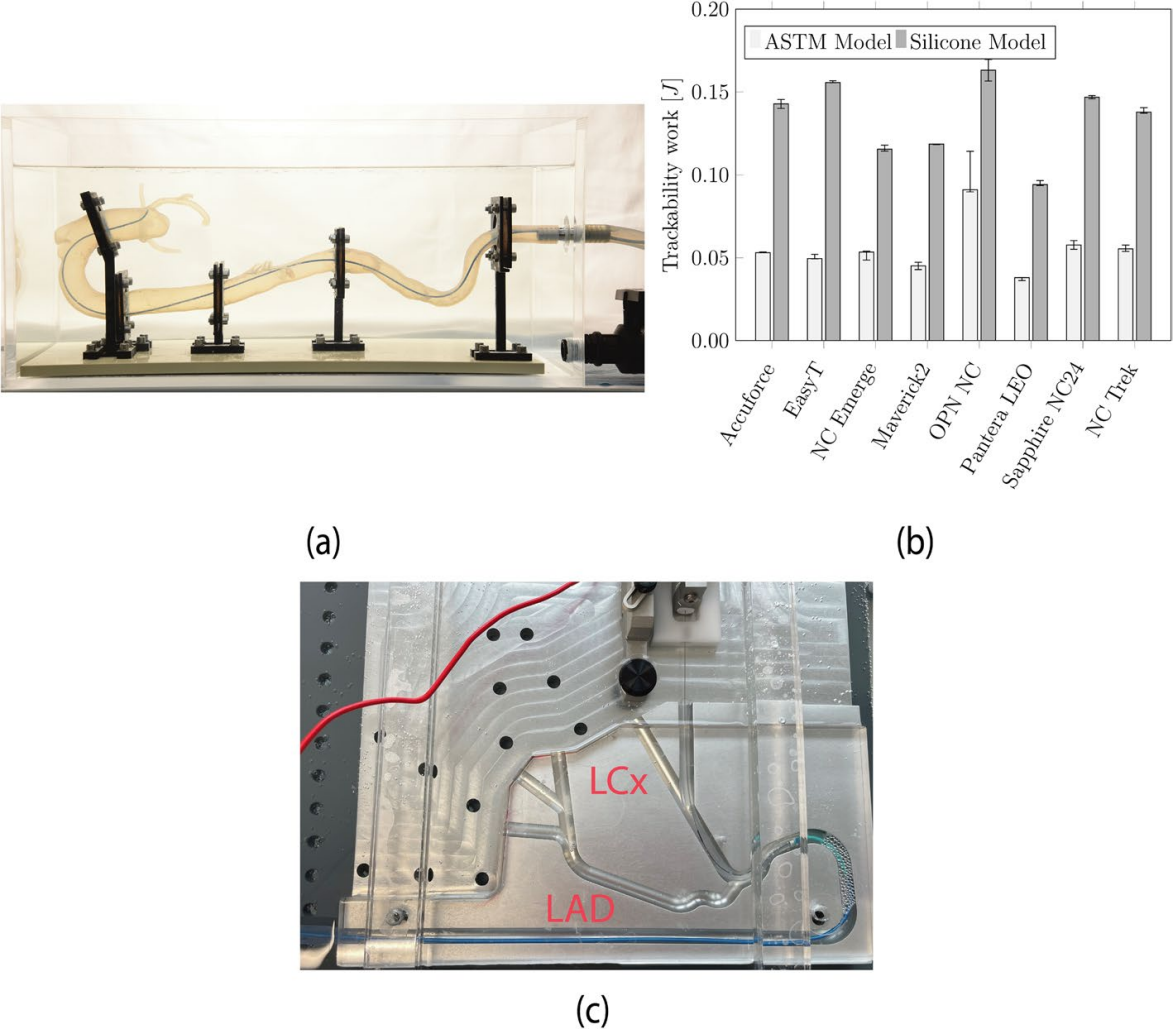
**a** The final result of the patient-specific silicone model is shown from the femoral artery to the coronary arteries. **b** Median results and IQR of the trackability test on commercial PTCA balloon catheters measured using the ASTM and the silicone model. **c** ASTM F2394 model showing the left anterior descending (LAD) and left circumflex (LCx) coronary arteries

The finalized 3DPSP is shown in Fig. 5a. The model is divided into five segments and placed inside a heatable water bath. A constant WT between 1 mm ($31.0 {\text{mm}}^{2}\cdot {\text{mmg}}^{-1}\cdot {10}^{3}$) and 2 mm ($14.7\cdot {\text{mm}}^{2}\cdot {\text{mmg}}^{-1}\cdot {10}^{3}$) is used for the coronary arteries. The WT was measured after each brushing to ensure accurate thickness. The trackability results in Fig. 5b indicate that the overall trackability work is greater in the silicone model. The OPN NC shows the highest trackability work of 0.16 J (0.013) in the silicone model and 0.091 (0.025) in the ASTM model; see Fig. 5c. The Pantera LEO exhibited the lowest trackability in both models, with 0.09 J (0.003) in the silicone model and 0.038 J (0.019) in the ASTM model.

## Discussion

### Mechanical properties

OCT measurements have proven to be suitable for evaluating the compliance of silicone tubes. The assumption of linearity within the physiological range for compliance calculation is acceptable, considering that the Pearson correlation coefficient for both uniform and stenosed silicone tubes was never lower than 0.968. Compliance can be adjusted through modifications to parameters such as the WT, Young’s modulus, or inner diameter. Furthermore, calcification in the form of gypsum was introduced. However, the difference from plain silicone is only significant for the first cycle of the radial tensile test. One reason is that some damage occurs in the calcified layer due to the high strains. In reality, the physiological strain should be approximately 10–30%. Therefore, the artery is not stretched to such a high degree. Furthermore, the addition of more gypsum could increase the material stiffness.

The reported compliance for healthy coronary arteries is $38.07 \frac{m{m}^{2}}{mmHg}\cdot {10}^{3}$. This value is comparable to the compliance of silicone tubes $\left(31.0 \frac{m{m}^{2}}{mmHg}\cdot {10}^{3}\right)$ with a WT of 1 mm and an inner diameter of 3 mm. The measurement of stenosed coronary arteries without calcification was reported to be $21.08 \frac{m{m}^{2}}{mmHg}\cdot {10}^{3}$, which is in good agreement with the compliance of stenosed silicone tubes without calcification and a WT of 2 mm $22.4 \frac{m{m}^{2}}{mmHg}\cdot {10}^{3}$.

To achieve stenosed coronary arteries with calcification, $18.18 \frac{m{m}^{2}}{mmHg}\cdot {10}^{3}$ silicone tubes with an inner diameter of 2.5 mm and a WT of 2 mm $16.5 \frac{m{m}^{2}}{mmHg}\cdot {10}^{3}$ or an inner diameter of 3 mm and a WT of 1.8 mm were used.

Nevertheless, notable scattering was evident in the measured values. Multiple factors contribute to this scattering. While OCT is renowned for its superior image resolution compared to IVUS [33], it is presumed that measurement errors stemming directly from the imaging technique are negligible. However, OCT measurement software automatically assesses the inner lumen area during postprocessing. The lumen wall may not be consistently recognized in certain instances, necessitating manual adjustments that could introduce errors.

Furthermore, inherent variations between specimens due to the manufacturing process may also contribute to differences. A significant eccentricity was noted, particularly in the stenosed specimens produced using a 3D-printed casting mold. This suggests that precise positioning of the core during manufacturing was challenging.

A relationship between Young’s modulus and compliance was shown. However, Young’s modulus required to obtain similar compliance as measured by OCT is much greater (110 kPa) than the measured modulus of 43–75 kPa.

This can be attributed to differences in the stress distribution between the radial tensile test and the cylinder under inner pressure. Furthermore, the strain measurement is much more precise when using the OCT method than the traverse of the tensile testing machine. Additionally, the pressure is applied uniformly on the vessel wall during the OCT measurement, causing uniform deformation. In contrast, in the tensile test, the force is only applied on 2 points, causing stress peaks. The clamps and pins are also deformed during the tensile test. A higher strain automatically results in a lower Young’s modulus. In the future, the strain should be measured using a video extensometer to increase the accuracy.

Furthermore, while the compliance measurements were performed statically, the radial tensile tests were performed quasistatically. Therefore, different strain rates are used, which might also influence the results and can explain differences in Young’s modulus.

ELASTOSIL Vario 15A was previously investigated by Illi et al. [26]. The obtained Young’s modulus of 183 kPa is comparable to that estimated by OCT (110 kPa).

Additionally, Young’s modulus was obtained from the radial tensile test. This test was chosen to be as close as possible to the final geometry instead of producing another test specimen geometry. Even though the radial tensile test, or ring pull test, is a uniaxial tensile test, the stress state is more complex than that in a typical dog-bone specimen [26] due to the stress concentration at the pins. There have been attempts to [34, 35] introduce correction factors to match the results of the radial tensile test to those of the uniaxial tensile tests, depending on the ratio between the pins used and the wall thickness. Even though the ratio is small in our case, it is still visible in Fig. 2 c) that the radial tensile tests show different behavior than the uniaxial tensile test and underestimate the stresses since the peak stresses are not considered, resulting in a reduced Young’s modulus.

The Young’s modulus of coronary arteries is reported to be between 0.55 and 4.11 MPa [36–38]. Similar to compliance, there is great variability in the reported values. The variability can be attributed to the coronary arteries, differences in the measured setup, and sample preparation. While most of the methods rely on extracted and cut coronary arteries, a value of 0.61 MPa was measured directly in the coronary arteries using OCT and a pressure sensor [39]. Therefore, this value is most comparable to the values obtained in this study. The agreement in compliance despite differences in Young's modulus can be attributed to the combined effects of geometry, material behavior, and the nature of the tests conducted. Compliance is influenced by both the material properties and the structural characteristics of the specimen, allowing different materials to exhibit similar compliance under certain conditions, even if their intrinsic stiffness differs. The wall thickness of coronary arteries is approximately 0.75 mm [40]. The ELASTOSIL Vario 15A was selected to avoid walls that are too thin and might fracture easily, which would be the case for a stiffer silicone (see supplementary material). The resulting wall thickness for the silicone was between 1 and 2 mm. This highlights the importance of considering material properties and structural factors when comparing biological tissues with synthetic materials.

Even though compliance is a widely used indicator for biological tissue, it has to be noted that it has some limitations when comparing arteries due to its dependency on artery size. Distensibility, another frequently cited parameter of biological tissue, characterizes the stress within arterial walls. It is calculated by dividing compliance by the initial cross-sectional area. Consequently, this value is independent of the initial cross-sectional area. However, if we assume similar cross sections and Young’s modulus but vary the WT, the distensibility decreases as the WT increases.

The comparison of the hyperelastic material model to the experimental data shows that even though the conversion from shore hardness to Young’s modulus is very simplified, good agreement can be achieved. The dependency on the deformation load should be considered for a more refined material model, and multiple constitutive laws should be compared.

### Friction

After correcting the *p*-value, all the methods tested, except for the use of glass beads and the PLL-g-PEG coating, significantly reduced the friction of the silicone. However, there are several drawbacks to these individual methods. Even though the glass beads are only embedded into the surface of the silicone and are made from several individual beads instead of a glass layer, Young’s modulus is greater than that of plain silicone. This can be attributed to a friction change between the steel cylinders and the silicone, which impacts the Young’s modulus. However, the difference was not significant (*p* > 0.05).

Both Parylene C coatings increase the Young’s modulus compared to plain silicone. This has to be considered when designing compliance measures. Additionally, samples with Parylene C coating show a decrease in Young’s modulus during later cycles. This could be because the Parylene C layer is too thin and becomes damaged when large strains are applied. This phenomenon becomes especially visible for the coating thickness of 1.2 µm. However, it must be noted that the applied strain during the radial tensile test is much greater than the physiological strain.

The potential impact of using soap or lubricant on the coating integrity of individual catheters remains uncertain. Further investigation of this effect will be necessary in the future. Additionally, applying either method to the model entails a significant time investment and necessitates post-use cleaning procedures. Given its low friction coefficient of 0.13, which closely aligns with reported values (0.046 [41] and 0.02–0.6 [42]), a lubricant (Durex, Perfect Glide, Silicone Based) was selected for the finalized model.

Despite the high costs, the PLL-g-PEG coating seems promising since it does not impact the mechanical properties, does not require post-use cleaning procedures, and, when achieving proper attachment to the silicone, it is assumed that it will not impact the coating of the catheters. However, this method cannot be studied further in the present work due to the limited material.

### 3DPSP

For the finalized model, a patient with a low degree of tortuosity of coronary arteries was selected to minimize the complexity. Despite the time-consuming manufacturing process, it was shown that the brushing method was a possible process for manufacturing complex geometries. However, further research is required to produce samples with greater reproducibility. In the future, tortuosity should be increased to mimic challenging cases. Furthermore, introducing stenotic regions can help study the crossability of catheters.

The current 3DPSP model can train PCI based on balloon catheter handling, stenting, and imaging techniques such as OCT and IVUS. The current model is too elastic for more complex devices, such as rotational or orbital atherectomy devices, since the working principle of rotablation involves grinding the calcified material while leaving the elastic tissue intact [43]. Even though the silicone is translucent, it is not as transparent as the ELASTOSIL RT601. Therefore, the use of image velocimetry is limited. Adaptation is required to connect the coronary arteries and the aorta to a flow loop to generate a pulsatile flow.

The manufacturing time for one set of the aortic arch and the coronary arteries is approximately 300 min. (The duration for exporting from the CT scan and 3D printing the core is not specified, as it varies depending on the geometries involved.) Due to the modular setup, different geometries can be exchanged and assessed efficiently within a practical timeframe. In Table 5, the main advantages and disadvantages of the manufacturing process are listed.

**Table 5:** Advantages and disadvantages of the proposed manufacturing process for a 3DPSP

Advantage	Disadvantage
Manufacturing with simple lab equipment (3D printer, polisher, vacuum pump, and scale)	The manual process depends on the experience of the manufacturer
No manufacturing and designing of casting molds	Close monitoring of the wall thickness during fabrication. Wall thickness variation along the length of the coronary artery is only possible to a limited extent. Material accumulation is possible
The mechanical properties of ELASTOSIL Vario 15 are closer to the ones of tissue compared to the screened 3D-printed material. The lumen can be directly obtained from the CT scan, and no step is required to generate the actual wall thickness in the CAD	Mechanical properties are only comparable in a certain pressure range (70–120 mmHg)
Low material costs (Filament for 3D printer and silicone)	Manufacturing requires a higher degree of personal labor compared to 3D printing
Tunable properties by varying the WT	Simplified tissue response
Modular design	
Resilience: silicone can withstand repeated use with limited degradation compared to currently available 3d printable elastic materials	

Based on the results of the trackability test, the trackability measured inside the silicone model is greater than that measured inside the ASTM model. This may be because the tortuosity of the coronary arteries and the tortuosity of the aorta itself influence catheter performance. The force required to advance the catheter is increased since the stiff hypotube must pass through the bend GC. Furthermore, the difference between the OPN NC and the other investigated catheters was less pronounced in the silicone model.

## Limitations

In addition to the limitations of the postprocessing of the OCT, the strain measurement for the radial tensile test, and the general difference between the radial tensile test and the uniaxial tensile test and the large scattering, another limitation of this study is the sample size. For each test, approximately 3–4 specimens were tested. More specimens would be required to increase the statistical significance. Furthermore, tensile tests were performed at room temperature.

A very basic hyperelastic material model was used, and the curve fit could only be performed by using available Young’s moduli or the experimental uniaxial tensile data. The dependency on different deformation modes could not be evaluated.

The chosen manufacturing process largely depends on the expertise of the individual producing the silicone part and the equipment used. No significant difference was observed between the cast samples (without acetone) and the brushed samples (with acetone). However, the potential impact of acetone used during demolding on the mechanical properties of silicone remains a possibility and warrants further investigation.

It should be noted that in the beginning, the PLL-g-PEG coating was lower. However, the friction increased after changing the water in the artery. Therefore, no proper attachment of the polymer brushes was achieved. Due to limited material, the test could not be repeated.

The mechanical behavior of the final model could not be directly verified. However, all tests were conducted on tubular specimens manufactured using the same process as the final model. Material accumulation may occur at the carina of the coronary artery or irregularities within the coronary lumen, potentially affecting mechanical behavior. Additionally, the extent to which the final model differs from the actual patient-specific geometry remains unverified due to the multiple processing stages, including CT scan extraction, CAD modeling, slicing, printing, and material polishing.

## Conclusion

This study presents a method to produce patient-specific silicone models with realistic mechanical and frictional properties. Models that mimic the desired characteristics were achieved using ELASTOSIL Vario 15 silicone and applying a brushing technique to a 3D-printed lost core. OCT measurements can be used to evaluate silicone tube compliance effectively. Despite Young's silicone modulus being lower than that of coronary arteries, the compliance of silicone tubes can be tuned to match the compliance of coronary arteries through modifications in wall thickness and consideration of the inner diameter. Furthermore, a relationship between compliance and Young’s modulus was shown, indicating limitations in coronary artery compliance and distensibility values due to the dependency on the actual arterial geometry. Although radial tensile tests are similar to the actual specimen geometry, the complex stress state limits their use for silicones. However, the scarcity of available literature and the interdependency among numerous parameters pose challenges in precisely tuning the parameters required to achieve the desired compliance.

## Methods

**Fig. 6:**
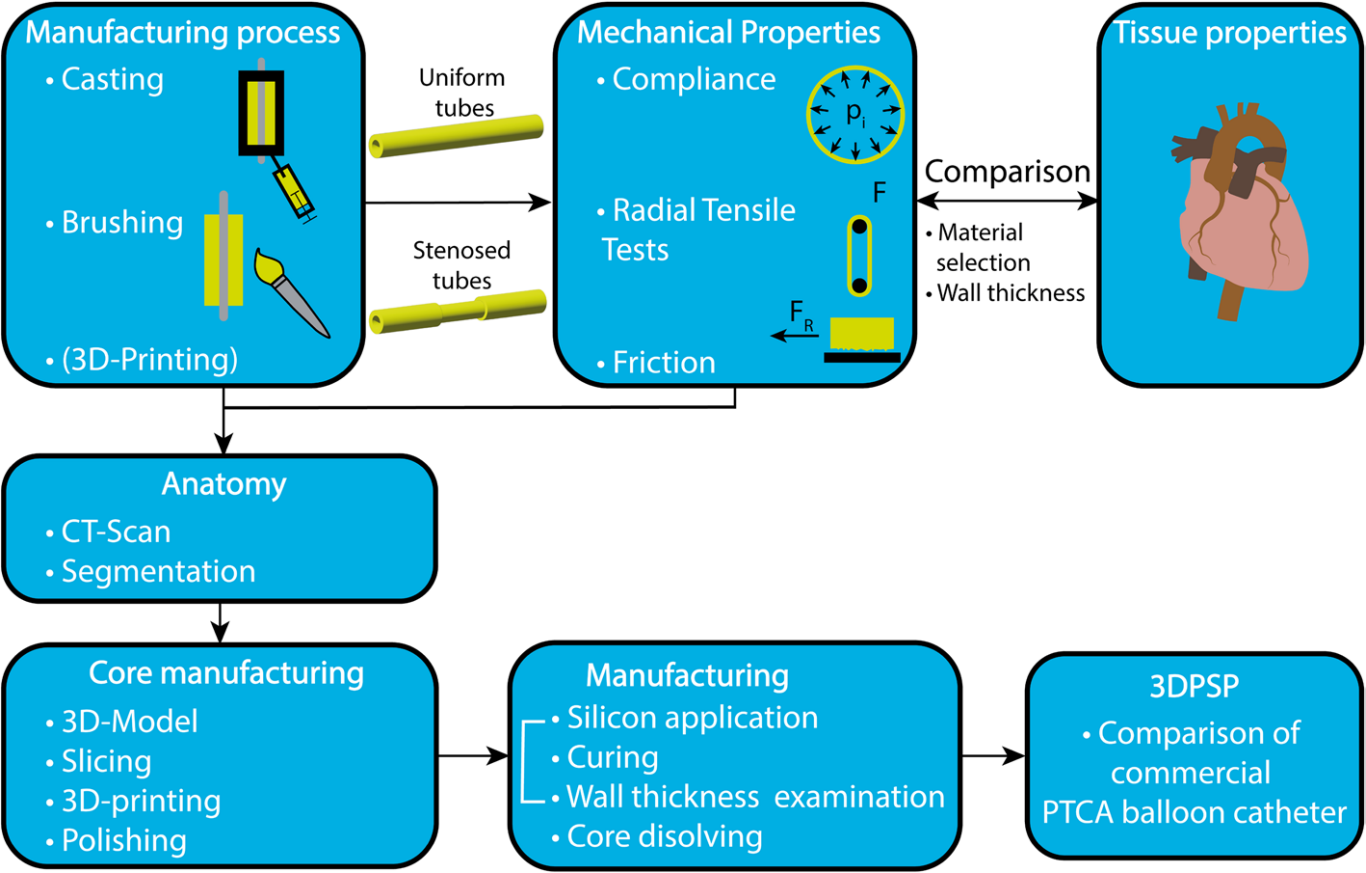
Development strategy for developing a 3DPSP for evaluating PTCA balloon catheters, from simplified specimens for the manufacturing process and parameter definition to the manufacturing process of the finalized 3DPSP

The following strategy was applied to develop the 3DPSP (see Fig. 6). Mimicking the mechanical properties of human tissues ensures that the phantom behaves realistically, providing accurate experimental outcomes. Stiffness is essential for replicating physiological conditions, and friction between tissue and medical devices is essential in real-life medical procedures. Incorporating frictional properties into the phantom ensures that the simulation closely mimics the tactile feedback and resistance encountered during actual interventions. By replicating anatomical features, the phantom can validate medical devices or procedures in a more representative environment.

### Materials

Different materials were screened before this investigation to evaluate materials with properties similar to literature values of human coronary arteries (see Table 6).

**Table 6:** Compliance values of coronary arteries are presented as the mean (SD) measured by IVUS and OCT

Imaging technique	Arterial classification	Compliance $\frac{m{m}^{2}}{mmHg}\cdot {10}^{3}$
IVUS [17–19]	Healthy coronary arteries Stenosed arteries without calcification Stenosed arteries with calcification	40.88 (20.12) [17], 17 (5) [18] 19.87 (11.13) [17], 17.57 (4.78) [19] 9.66 (6.34) [17], 12 (2) [18]
OCT [20]	Healthy coronary arteries Stenosed arteries without calcification Stenosed arteries with calcification	38.07 (17.45) 21.08 (16.04) 18.18 (17.45)

Intravascular ultrasound (IVUS) [17–19] and optical coherence tomography (OCT) [20] were employed in a clinical setting to measure coronary artery compliance in living subjects based on the change in cross-sectional area due to physiological pulse pressure. The IVUS measurements align with results from other studies for stenosed arteries. For healthy and non-calcified coronary arteries, the mean values are consistent between IVUS and OCT. However, there is a greater deviation between the two methods for stenosed arteries with calcification. Most measurements exhibit a large standard deviation (SD), highlighting significant patient variability.

An overview of the investigated materials and their corresponding results can be found in the supplementary material. Based on these results, ELASTOSIL Vario 15A (Wacker Chemie AG, Munich, Germany) was used during this work. Furthermore, stewalin (Glorex, Fuellinsdorf, Switzerland) was added to the stenosed tubes for calcification.

The median and interquartile range (IQR) were calculated for all tests. Statistical tests were performed with R version 4.3.2 in RStudio 2023.03.0 (Posit Software, PBC, Boston, Massachusetts, USA). The p-values were calculated using the Wilcoxon signed-rank test. A *p*-value of <  = 0.05 was considered significant.

### Manufacturing process

Before manufacturing the 3DPSP, simple tubular specimens were used to determine the wall thickness and properties that matched those of coronary arteries. The process for producing these specimens is detailed below.

In the first manufacturing step, the silicone was hand-mixed and degassed for 10 min at 10 bar to eliminate air bubbles.

#### Casting

Uniform silicone tubes with a constant inner diameter, representative of the luminal opening, and a constant outer diameter, representative of healthy coronary arteries, were produced based on the casting mold procedure. Three different casting molds produced tubes with wall thicknesses (WTs) of 0.5 mm, 1.0 mm, or 2.0 mm, depending on the outer part of the mold. The inner diameter was formed by a metal rod, which had a diameter of 3.0 mm. For the demolding of the finished part, the outer part is designed with two half shells. More information can be found in the supplementary material.

The manufacturing of a stenosed silicone tube is based on the principle of lost core casting. Lost core casting is a two-part system comprising a destructible ‘‘male’’ core and a negative ‘‘female’’ mold. The male core represents the inner lumen of a blood vessel, and the female mold represents the outer wall of a blood vessel. A circular dog bone shape was used for these specimens, with stenosis in the center. A healthy left coronary artery has an initial diameter of approximately 2.0–5.5 mm [21, 22]. Therefore, a diameter of 5 mm was chosen for the healthy segment of the core design. To simulate 75% luminal stenosis, narrowing to a diameter of 2.5 mm was introduced at the center of the core, resulting in a minimum lumen area (MLA) of 4.9 mm^2^ The core was designed using SolidWorks 2019 (Waltham, Massachusetts, USA) and was 3D printed using an Ultimaker S5 (Ultimaker, Utrecht, Netherlands). The destructible core was printed using PolySmooth™ (Polymaker, Shanghai, China) and smoothed in isopropanol mist to remove the layer lines created during 3D printing using Polysher™ (Polymaker, Shanghai, China). The stenosis was introduced by increasing the material thickness or adding gypsum to the silicone at a ratio of 1:2 (double the amount of silicone).

#### Brushing

In addition to casting, silicone tubes were manufactured by repeatedly brushing a thin silicone layer onto a core. For this process, a core with a constant diameter of 3.0 mm was 3D printed from Polysmooth^™^ and smoothed. A thin silicone layer was brushed on the core and then cured at 50 °C for 10 min. The brush-curing process was repeated until the desired WT was reached. Approximately 15 brushing layers were necessary to reach a WT of 1.5 mm. Finally, the molds were dissolved in a bath of acetone for 3 h.

### Mechanical properties of the materials

#### Compliance measurement

OCT measurements were utilized to assess sample compliance. A Dragonfly™ OPTIS™ Imaging Catheter (Abbott, Chicago, Illinois, USA) was used. OCT measurements can be performed using an automatic or stationary pullback method. The probe is withdrawn through the blood vessel over a certain distance at 20 mm/s in the automatic pullback method. Notably, the distance and speed of an OCT pullback can vary between measurements. During automatic pullback, which helps assess a stenotic region, multiple images are generated and subsequently stacked to form a longitudinal cross-sectional blood vessel profile. In the stationary measurement, the probe remains in place, capturing images of the same cross section for a specified duration. During this research, the stationary OCT method was utilized to identify transverse cross-sectional changes in the inner lumen, ultimately determining each specimen's cross-sectional compliance and geometrical shape. The equipment required for the measurements included an OCT console with a light source, a compatible catheter, a pressure transducer, and an adjustable manual pump.

The measurements were performed in a water bath at 37 °C. The pressure within each test phantom was gradually increased by 10 mmHg using an indeflator (SIS Medical AG, Frauenfeld, Switzerland). Cross-sectional snapshots were taken at each pressure setpoint. The pressure was recorded in LabView 2019 (National Instruments, Austin, Texas, USA) with an MLT844 physiological pressure transducer (AD Instruments, Sydney, Australia). The cross-sectional compliance is defined as per Eq. 1. [23]:

1 $$CC=\frac{\Delta A}{\Delta P}\cdot {10}^{3}\left[\frac{m{m}^{2}}{mmHg}\right],$$

where ΔA is the change in cross-sectional area proportional to the change in pressure ΔP. The compliance was identified in the range of 70 mmHg to 130 mmHg, reflecting the average physiological blood pressure. The age-standardized average diastolic and systolic blood pressures for men and women were 78.7 and 127.0 mmHg and 76.7 and 122.3 mmHg, respectively [24]. Linear behavior between the measured cross section and the pressure was assumed and assessed for each specimen using Pearson’s linear correlation coefficient $r$.

By expressing the change in cross-sectional area with the change in radius $\Delta r$, the following equation (Eq. 2) for compliance can be obtained, where ${r}_{i}$ is the lumen radius under initial or lower pressure conditions:

2 $$CC=\frac{\pi \cdot \Delta {r}^{2}+\pi \cdot 2\cdot {r}_{i}\cdot \Delta r}{\Delta P}\cdot {10}^{3}\left[\frac{m{m}^{2}}{mmHg}\right].$$

Therefore, the radius change $\Delta r$ can be expressed by Eq. 3:

3 $$\Delta r=-{r}_{i}+\sqrt{{r}_{i}^{2}+\frac{CC\cdot {10}^{-3}\cdot \Delta P}{\pi }}\left[mm\right].$$

When assuming linear elastic behavior of the silicone phantoms and a rotational symmetric cross section, the following equation (Eq. 3) for closed tubes under inner pressure $\Delta r$ can be calculated using Eq. 4 [25]:

4 $$\Delta r=\frac{{p}_{i}\cdot {r}_{i}^{3}}{E\cdot ({r}_{a}^{2}-{r}_{i}^{2})}\cdot \left[\frac{{r}_{a}^{2}}{{r}_{i}^{2}}\cdot \left(1+\mu \right)+1-2\cdot \mu \right]\left[mm\right],$$

where ${r}_{a}$ is the outer radius, $E$ is the Young’s modulus, and $\mu$ is the Poisson’s ratio. For silicone, an incompressible material is assumed, and therefore, $\mu =0.5$. Young’s modulus can express compliance, and vice versa, using Eqs. 3, 4.

The repeatability of the measurement was investigated by performing multiple measurements on the same stenosed silicone tube under the same conditions. These results are shown in the supplementary material.

#### Radial tensile test

**Fig. 7:**
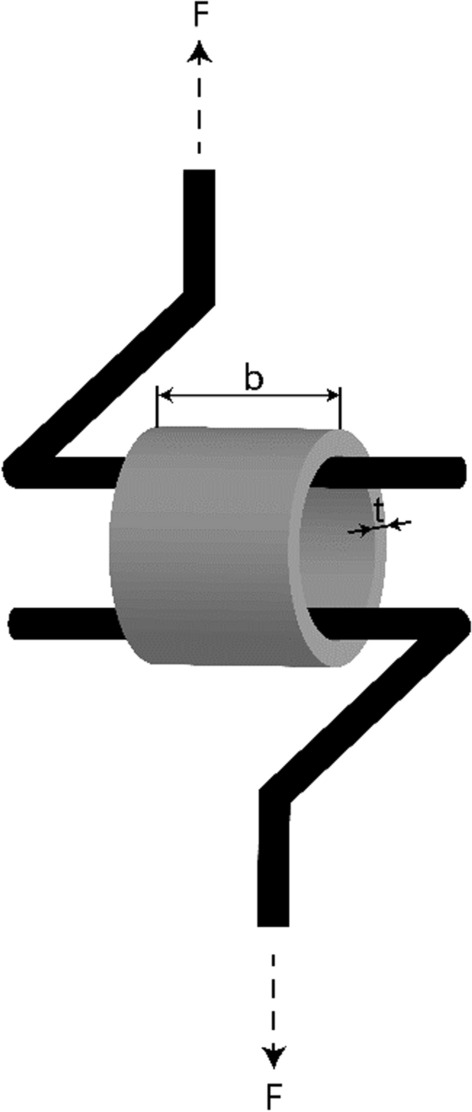
Measurement of the radial tensile properties

To measure the circumferential stress and strain behavior of the samples, a radial tensile test (see Fig. 7) is used. The tests were performed using a Shimadzu AGS-X 10 kN (Shimadzu Corporation, Kyoto, Japan). The force $F$ was recorded using a 200 N load cell (accuracy of the measured value of ± 0.5%). The displacement $d$ was recorded by crosshead position detection (accuracy ± 0.1% indicated value or ± 0.01 mm). The silicone tubes were cut into 15 mm segments. Two hardened steel cylinders with a diameter of 1.4 mm were inserted inside the sample 0.2 mm from each other. These cylinders were fixed to aluminum supports, which were clamped into the jaws of the tensile test machine. This allows sufficient rigidity of the test support.

For the tensile test experiment, a specified load pattern was used to stretch the samples radially.

Initially, a preload of 0.05 N was applied. Subsequently, the specimen underwent five loading–unloading cycles wherein the force was incrementally increased to 5 N at a rate of 25 mm/min and decreased to 0 N at 50 mm/min. Following this, another set of five loading–unloading cycles with the same velocities was performed, with the force reaching 7.5 N.

The corresponding tensile stress $\sigma$ was calculated using the formula in Eq. 5:

5 $$\sigma =\frac{F}{2tb} \left[\frac{N}{m{m}^{2}}\right],$$

where *F* is the measured force, $t$ is the WT, and $b$ is the length of the test specimen.

The tensile strain $\varepsilon$ was calculated using the formula in Eq. 6:

6 $$\varepsilon =\frac{\Delta d}{d} \left[-\right],$$

where Δd is the measured stroke and *d* is the length of the original configuration, which is the initial inner diameter of the specimen.

The Young’s modulus *E* was then calculated based on Eq. 7 using the least squares linear regression method:

7 $$E=\frac{\Delta \sigma }{\Delta \varepsilon } \left[kPa\right].$$

Silicones usually exhibit hyperelastic behavior. Therefore, the material is usually described by models that can account for nonlinear behavior rather than assuming linear elasticity. The strain energy function $W$ describes the relationship between stress and strain for these materials. Common hyperelastic material constitutive models include the Ogden, Arruda-Boyce, polynomial models, and variants of polynomial models such as the Mooney-Rivlin, neo-Hookean, and Yeoh models. The input of experimental data from uniaxial, biaxial, pure shear tensile, and confined compression tests is required [27] to derive such hyperelastic material constitutive models. However, basic material models such as the neo-Hookean model (see Eq. 8) can already be defined by the shear modulus $G$ and the bulk modulus $K$ obtained from the uniaxial tensile test or by performing a model curve fitting to the experimental data from the uniaxial tensile test. Since no classic uniaxial tensile test was performed in this study, this study relied on data from Illi et al. [26] for curve fitting.

8 $$W={C}_{10}\left({I}_{1}-3\right)+\frac{1}{{d}_{1}}{\left(J-1\right)}^{2} ,$$

where ${C}_{10}$ and ${d}_{1}$ are material parameters that can be approximated by ${d}_{1}=\frac{2}{K}$ and ${C}_{10}=\frac{G}{2}$ [27], ${I}_{1}$ is the first strain invariant of the right Cauchy–Green deformation tensor, and J is the total volumetric ratio, which is 1 for an incompressible material.

The shear modulus $G$ and bulk modulus $K$ can be converted from Young’s modulus by Eq. 9 and Eq. 10, respectively:

9 $$G=\frac{E}{2\left(1+\mu \right)} \left[MPa\right],$$

10 $$K=\frac{E}{3\left(1-2\mu \right)} \left[MPa\right].$$

As mentioned above, a Poisson’s ratio $\mu$ of 0.5 is assumed. This results in an infinite value for $K,$, and in addition, ${d}_{1}$ becomes zero.

There have been attempts in the literature to transform the shore hardness of rubbery materials directly into Young’s modulus [28–30]. The conversion from shore hardness $S$ to Young’s modulus $E$ is shown in Eq. 11 [30]:

11 $${E}_{Rigbi}={e}^{\frac{S-35.22735}{18.75487}} \left[MPa\right].$$

#### Frictional properties

After manufacturing, the silicone features a highly sticky surface, making the insertion of catheters nearly impossible. For this evaluation, specimens were manufactured using the brushing approach described above and then tested under the following conditions:

- Filling with deionized water.
- Silicone with embedded glass beads 125–200 µm in size and filled with deionized water.
- Parylene C coating with a thickness of 1.2 µm (COAT-X SA, La Chaux-de-Fonds, Switzerland), filled with deionized water.
- Parylene C coating with a thickness of 6.25 µm (COAT-X SA, La Chaux-de-Fonds, Switzerland), filled with deionized water.
- Poly(L-lysine)-graft-poly(ethylene glycol) (PLL(20)- g[3.5]-PEG(5)) [31] and filled with deionized water.
- The samples were filled with a deionized water–soap mixture (10:1 ratio).
- Filled with a lubricant (Durex, Perfect Glide, Silicone Based).

Further information on the tests performed, and the methods used to reduce friction can be found in the supplementary material. Five specimens with a WT of 1.5 mm and an inner diameter of 3 mm were produced for each of the abovementioned methods. The WT and length of the manufactured samples were measured using a Nikon SMZ745T stereo Microscope (Nikon Instruments Inc., Tokyo, Japan). Friction was evaluated by measuring the friction between the coating and a polyamide 12 (PA12) tube using the device described in [32]. Further information on the tests performed and the coating manufacturing can be found in the supplementary material. Furthermore, the influence of the coating on the mechanical properties was tested using the radial tensile test for five cycles until a force of 5 N was reached.

### 3DPSP manufacturing

**Fig. 8:**
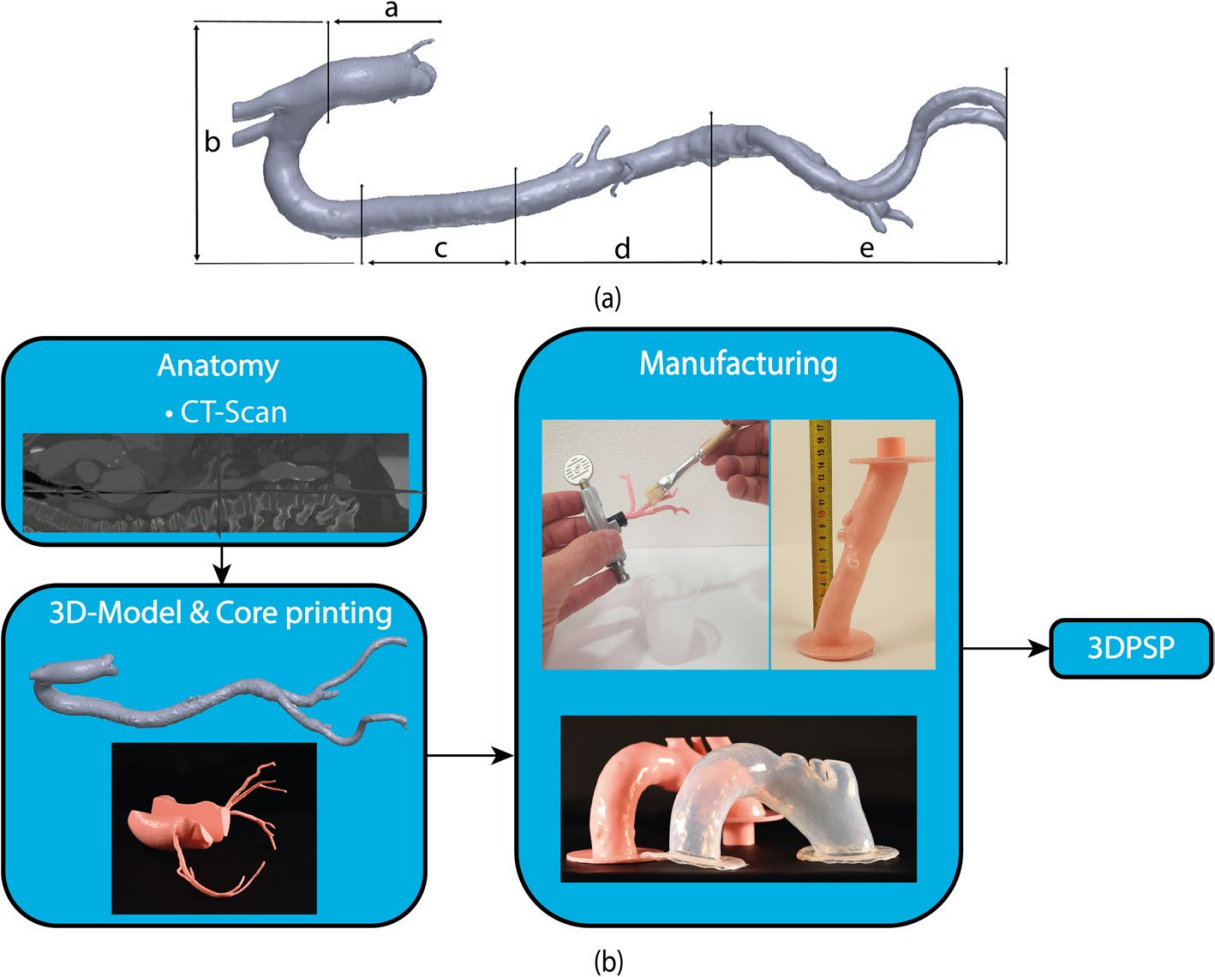
**a** Entire core for the 3DPSP containing the individual segments from the aortic root to the femoral artery. **b** Manufacturing steps to produce the final 3DPSP

The femoral artery to the aortic root was segmented from the computed tomography (CT) scan of a 72-year-old male. The aortic root and the coronary arteries were segmented from a CT scan of a 52 year-old male. The open-source software 3D slicer (The Slicer Community, Earth, Texas, United States) and Meshmixer (Autodesk, San Francisco, California, United States) were used for segmentation. The model (see Fig. 8a) was divided into the following segments: **a** the aortic root with the coronary arteries positioned on the balloon, **b** the aortic arch, **c** the descending aorta, **d** the renal aortic part, and **e** the right femoral artery to enable a modular setup. The final design was performed in SolidWorks 2019 (Dassault Systemes, Waltham, Massachusetts, United States).

The generation of casting molds was challenging due to the tortuosity and unsymmetrical shape of the arteries. Therefore, the brushing approach was used. The segments were 3D printed from PolySmooth^™^. Water-dissolvable polyvinyl alcohol (PVA, Ultimaker) was used as a support structure. Before applying the silicone, the support structure was dissolved in water, and the PolySmooth™ was smoothed. An example of a smoothed core of the aortic root with coronary arteries is shown in Fig. 8b. The model was split and reassembled by dissolving the surface between the halves with acetone for better printing quality and less support material. The overall fabrication process is shown in Fig. 8b.

### Comparison of commercial PTCA balloon catheters

Measurements of trackability were performed on an IDTE 1000 track tester (MSI, Phoenix, Arizona, USA). Tests were performed on a 2D track model (ASTM F2394) and the 3D silicone model developed in the present work. First, the inside of the silicone model was filled with a mixture of deionized water and soap to facilitate the insertion of the 5F JL 4.0 guide catheter (GC) (Medtronic, Dublin, Ireland). The GC was inserted into the silicone model, pushed through the descending aorta and the aortic arch, and placed at the ostium of the left anterior descending (LAD) artery. Furthermore, an ASAHI SION blue straight guide wire (GW) (ASAHI INTECC Co., Ltd., Tokyo, Japan) was advanced through the GC into the LAD artery. After each test, a syringe was used to fill the coronary artery with 1 ml of the lubricant mentioned above from the distal end.

The PTCA balloon is pushed along the GW until the tip is at the ostium of the LAD artery. The force ${F}_{track}$ required to move the catheter along the LAD was measured. Additionally, the insertion depth was recorded. After 7 cm of insertion, the catheter was retracted. The resulting insertion forces of the catheter models are compared against each other. The same settings are used for both models.

Furthermore, the area under the curve, which defines the work required for insertion and extraction, was evaluated. The tests were carried out in a water bath at 37 °C. The water was heated using an ANOVA Precision Cooker (Anova Applied Electronics, Inc., San Francisco, CA, USA) with an accuracy of ± 0.5 °C.

### Supplementary Information

Supplementary material 1.

## Data Availability

The datasets used and/or analyzed during the current study are available from the corresponding author upon reasonable request.

## Abbreviations

CT: Computed tomography
GC: Guide catheter
GW: Guide wire
IVUS: Intravascular ultrasound
IQR: Interquartile range
LAD: Left anterior descending (artery)
MLA: Minimal lumen area
OCT: Optical coherence tomography
PCI: Percutaneous coronary intervention
PTCA: Percutaneous transluminal coronary angioplasty
PA12: Polyamide 12
PLL-g-PEG: Poly(L-lysine)-graft-poly(ethylene glycol)
PVA: Poly vinyl-alcohol
WT: Wall thickness
3DPSP: 3D patient-specific phantoms
3D: Three dimensional

## References

[1] Van Den Haak L, Alleblas C, Rhemrev JP, Scheltes J, Nieboer TE, Jansen FW. Human cadavers to evaluate prototypes of minimally invasive surgical instruments: a feasibility study. Technol Heal Care. 2017;25:1139–46. 10.3233/THC-171029 28946605

[2] Petersen JL, Levin DB, Hou VW, Hagen CV, Anderson G, Block J, et al. Intact and isolated human cadaveric coronary artery perfusion models to facilitate research and education regarding coronary anatomy and pathology. J Invasive Cardiol. 2019;31:272–7. 31199349 10.25270/jic/19.3109.272

[3] Cesarovic N, Lipski M, Falk V, Emmert MY. Animals in cardiovascular research. Eur Heart J. 2020;41:200–3. 31909425 10.1093/eurheartj/ehz933

[4] Ohayon J, Finet G, Pettigrew RI. Biomechanics of coronary atherosclerotic plaque. Biomech Coron Atheroscler Plaque From Model to Patient. 2021;1:1–653.

[5] Gisterå A, Ketelhuth DFJ, Malin SG, Hansson GK. Animal models of atherosclerosis-supportive notes and tricks of the trade. Circ Res. 2022;130:1869–87. 35679358 10.1161/CIRCRESAHA.122.320263

[6] Cantor WJ, Lazzam C, Cohen EA, Bowman KA, Dolman S, Mackie K, et al. Failed coronary stent deployment. Am Heart J. 1998;136:1088–95. 9842025 10.1016/S0002-8703(98)70168-1

[7] Hh H, Fh J, Kk L, Jk T, Yw O, Pj O. Deliverability of integrity coronary stents in severely tortuous coronary arteries: a preliminary experience. J Invasive Cardiol. 2012;24:650–4. 23220980

[8] Bernhard B, Illi J, Gloeckler M, Pilgrim T, Praz F, Windecker S, et al. Imaging-based, patient-specific three-dimensional printing to plan, train, and guide cardiovascular interventions: a systematic review and meta-analysis. Hear Lung Circ. 2022;31:1203–18. 10.1016/j.hlc.2022.04.052 35680498

[9] Ormiston JA, Kassab G, Finet G, Chatzizisis YS, Foin N, Mickley TJ, et al. Bench testing and coronary artery bifurcations: a consensus document from the European bifurcation club. EuroIntervention. 2018;13:e1794–803. 29131803 10.4244/EIJ-D-17-00270

[10] Watanabe H, Saito N, Tatsushima S, Tazaki J, Toyota T, Imai M, et al. Patient-specific three-dimensional aortocoronary model for percutaneous coronary intervention of a totally occluded anomalous right coronary artery. J Invasive Cardiol. 2015;27:E139–42. 26136288

[11] Niizeki T, Iwayama T, Kumagai Y, Ikeno E, Saito N, Kimura T. Preprocedural planning using a three-dimensional printed model for percutaneous coronary intervention in an anomalous coronary artery. Am J Case Rep. 2020;21:1–5. 10.12659/AJCR.923007 PMC12571015 32305993

[12] Gach O, Finianos L, Palmers PJ, Testaguzza M, Ungureanu C. Complex percutaneous coronary intervention assisted by 3-dimensional printing model. JACC Cardiovasc Interv. 2022;15:e159–61. 35798492 10.1016/j.jcin.2022.04.029

[13] Brunette J, Mongrain R, Tardif JC. A realistic coronary artery phantom for particle image velocimetry: featuring injection-molded inclusions and multiple layers. J Vis. 2004;7:241–8. 10.1007/BF03181639

[14] Yazdi SG, Geoghegan PH, Docherty PD, Jermy M, Khanafer A. A review of arterial phantom fabrication methods for flow measurement using PIV techniques. Ann Biomed Eng. 2018;46:1697–721. 29987543 10.1007/s10439-018-2085-8

[15] Finn R, Morris L. An experimental assessment of catheter trackability forces with tortuosity parameters along patient-specific coronary phantoms. Proc Inst Mech Eng Part H J Eng Med. 2016;230:153–65. 10.1177/0954411915623815 26721906

[16] Biglino G, Verschueren P, Zegels R, Taylor AM, Schievano S. Rapid prototyping compliant arterial phantoms for in-vitro studies and device testing. J Cardiovasc Magn Reson. 2013;1:15. 10.1186/1532-429X-15-2 PMC3564729 23324211

[17] Alfonso F, Macaya C, Goicolea J, Hernandez R, Segovia J, Zamorano J, et al. Determinants of coronary compliance in patients with coronary artery disease: an intravascular ultrasound study. J Am Coll Cardiol. 1994;23:879–84. 10.1016/0735-1097(94)90632-7. 8106692 10.1016/0735-1097(94)90632-7

[18] Shaw JA, Kingwell BA, Walton AS, Cameron JD, Pillay P, Gatzka CD, et al. Determinants of coronary artery compliance in subjects with and without angiographic coronary artery disease. J Am Coll Cardiol. 2002;39:1637–43. 10.1016/S0735-1097(02)01842-9. 12020491 10.1016/S0735-1097(02)01842-9

[19] Williams MJA, Stewart RAH, Low CJS, Wilkins GT. Assessment of the mechanical properties of coronary arteries using intravascular ultrasound: an in vivo study. Int J Card Imaging. 1999;15:287–94. 10517378 10.1023/A:1006279228534

[20] Adnan KA, Robinson C, Biggs MJ, Morgan B, Rutty GN, Borsen A, et al. P2357Measurement of coronary artery compliance and stiffness index with novel application of optical coherence tomography in re-pressurised cadaveric coronary arteries. Eur Heart J. 2017. 10.1093/eurheartj/ehx502.P2357. 10.1093/eurheartj/ehx502.P2357

[21] Faletra FF, Pandian NG, Ho SY. Anatomy of the heart by multislice computed tomography. Anat Hear by Multislice Comput Tomogr. 2009. 10.1002/9781444300550. 10.1002/9781444300550

[22] Waller BF, Orr CM, Slack JD, Pinkerton CA, Van Tassel J, Peters T. Anatomy, histology, and pathology of coronary arteries: a review relevant to new interventional and imaging techniques—part I. Clin Cardiol. 1992;15:451–7. 1617826 10.1002/clc.4960150613

[23] Tozzi P, Corno A, Hayoz D. Definition of arterial compliance. Am J Physiol Circ Physiol. 2000;278:H1407–H1407. 10.1152/ajpheart.2000.278.4.H1407 10787279

[24] Zhou B, Bentham J, Di Cesare M, Bixby H, Danaei G, Cowan MJ, et al. Worldwide trends in blood pressure from 1975 to 2015: a pooled analysis of 1479 population-based measurement studies with 19·1 million participants. Lancet. 2017;389:37–55. 27863813 10.1016/S0140-6736(16)31919-5 PMC5220163

[25] Holzmann G, Meyer H, Schumpich G. Technische mechanik festigkeitslehre. Wiesbaden: Vieweg+Teubner Verlag; 2012.

[26] Illi J, Ilic M, Stark AW, Amstutz C, Burger J, Zysset P, et al. Mechanical testing and comparison of porcine tissue, silicones and 3D-printed materials for cardiovascular phantoms. Front Bioeng Biotechnol. 2023. 10.3389/fbioe.2023.1274673. 10.3389/fbioe.2023.1274673 PMC10725245 38107617

[27] Lalo D, Greco M. Rubber bushing hyperelastic behavior based on shore hardness and uniaxial extension. 24th ABCM Int Congr Mech Eng December 3–8, 2017, Curitiba, RP, Brazil. 2018.

[28] Kunz J, Studer M. Determining the modulus of elasticity in compression via the shore a hardness theoretical background. Kunststoffe Int. 2006;96:92–4.

[29] Meththananda IM, Parker S, Patel MP, Braden M. The relationship between Shore hardness of elastomeric dental materials and Young’s modulus. Dent Mater. 2009;25:956–9. 19286248 10.1016/j.dental.2009.02.001

[30] Ucar H, Basdogan I. Dynamic characterization and modeling of rubber shock absorbers: a comprehensive case study. J Low Freq Noise Vib Act Control. 2018;37:509–18. 10.1177/1461348417725954

[31] Aebischer P, Caversaccio M, Wimmer W. Fabrication of human anatomy-based scala tympani models with a hydrophilic coating for cochlear implant insertion experiments. Hear Res. 2021;404:108205. 10.1016/j.heares.2021.108205. 33618163 10.1016/j.heares.2021.108205

[32] Blossa P, Wolfgang R, Peter W, Christian W, Alexander R, Georg Dieter K, et al. Investigations of the pushability behavior of cardiovascular angiographic catheters. Biomed Mater Eng. 2003;13:327–43. 14646048

[33] Schmitt JM. Optical coherence tomography (OCT): a review. IEEE J Sel Top QUANTUM Electron. 1990;5:1205–15. 10.1109/2944.796348

[34] Mahutga RR, Schoephoerster CT, Barocas VH. The ring-pull assay for mechanical properties of fibrous soft tissues—an analysis of the uniaxial approximation and a correction for nonlinear thick-walled tissues. Exp Mech. 2021;61:53–66. 33583946 10.1007/s11340-020-00623-3 PMC7880234

[35] Van Haaften EE, Van Turnhout MC, Kurniawan NA. Image-based analysis of uniaxial ring test for mechanical characterization of soft materials and biological tissues. Soft Matter Royal Soc Chem. 2019;15:3353–61. 10.1039/C8SM02343C 30924833

[36] Ozolanta I, Tetere G, Purinya B, Kasyanov V. Changes in the mechanical properties, biochemical contents and wall structure of the human coronary arteries with age and sex. Med Eng Phys. 1998;20:523–33. 9832028 10.1016/S1350-4533(98)00050-2

[37] Karimi A, Navidbakhsh M, Shojaei A, Faghihi S. Measurement of the uniaxial mechanical properties of healthy and atherosclerotic human coronary arteries. Mater Sci Eng. 2013;33:2550–4. 10.1016/j.msec.2013.02.016. 10.1016/j.msec.2013.02.016 23623067

[38] Karimi A, Rahmati SM, Sera T, Kudo S, Navidbakhsh M. A combination of experimental and numerical methods to investigate the role of strain rate on the mechanical properties and collagen fiber orientations of the healthy and atherosclerotic human coronary arteries. Bioengineered. 2017;8:154–70. 27588460 10.1080/21655979.2016.1212134 PMC5398577

[39] Potlov AY, Proskurin SG, Frolov SV. Young’s Modulus Evaluation for the Blood Vessel Walls using Intravascular Optical Coherence Tomography. 2020;1–4.

[40] Fayad ZA, Fuster V, Fallon JT, Jayasundera T, Worthley SG, Helft G, et al. Noninvasive in vivo human coronary artery lumen and wall imaging using black-blood magnetic resonance imaging. Circulation. 2000;102:506–10. 10920061 10.1161/01.CIR.102.5.506

[41] Takashima K, Shimomura R, Kitou T, Terada H, Yoshinaka K, Ikeuchi K. Contact and friction between catheter and blood vessel. Tribol Int. 2007;40:319–28. 10.1016/j.triboint.2005.10.010

[42] Lin C, Kaper HJ, Li W, Splinter R, Sharma PK. Role of endothelial glycocalyx in sliding friction at the catheter-blood vessel interface. New York: Nature Publishing Group; 2020. 10.1038/s41598-020-68870-x. 10.1038/s41598-020-68870-x PMC7366638 32678286

[43] V Pj 2022 S N. Rotational Atherectomy. StatPearls Publ MA D.

