# Supplementary material for: Development of a patient-specific model of the human coronary system for percutaneous transluminal coronary angioplasty balloon catheter training and testing
Source: Biomed Eng Online. 2024 Aug 30;23:89. doi: 10.1186/s12938-024-01271-7 (PMC11363638; doi:10.1186/s12938-024-01271-7)
Supplement: Supplementary file 1 — Supplementary material 1. [file 12938_2024_1271_MOESM1_ESM.docx]

# Supplementary material

## Material screening

The following materials (see Table 1A), with varying Shore hardness and manufacturing methods, were investigated and compared to the literature values for coronary arteries:

**Table 1A.** Materials used during the material screening.

| **Material** | **Supplier** | **Shore**  **Hardness** | **Manufacturing Process** |
| --- | --- | --- | --- |
| Agilus 30 clear | Stratasys, Eden Prairie, Minnesota, USA | 30 A | PolyJet 3D Printing |
| Tango Plus Clear | Stratasys, Eden Prairie, Minnesota, USA | 37 A | PolyJet 3D Printing |
| Ecoflex | SmoothOn, Macungie, Pennsylvania, USA | 00-30 | Casting |
| ELASTOSIL RT601 | Wacker Chemie AG, Munich, Germany | 45 A | Casting |
| ELASTOSIL Vario | Wacker Chemie AG, Munich, Germany | 15, 20 and 30 A | Casting |

Three tubes were fabricated by the casting process described above from each material with a diameter of 0.5 mm, 1 mm, and 2 mm WT. Due to the very soft behavior of Ecoflex 00-30, instead of 0.5 mm WT, a 3 mm long lumen inside a silicone block with a thickness of 6 cm was used for this material. OCT was used to measure the compliance of each tube according to the method described above (Figure 1A).

Compliance is reduced for all materials when the WT increases. Due to fractured samples, not all the WTs of the 3D-printed Agilus 30 clear and Tango Plus clear materials could be tested. Due to the issues with fractured samples, these materials were considered unsuitable. Ecoflex 00-30 compliance was the highest of all the tested materials (1 mm: 292.5; 2 mm: 123.5 mm). The ELASTOSIL RT601 shows the lowest compliance. Compared to the values from the literature, the ELASTOSIL RT 601 is deemed unsuitable since WTs smaller than 0.5 mm are not feasible with the manufacturing method. The ELASTOSIL Vario 15A agreed best with the literature values for human coronary arteries and the measured values. The values for Ecoflex 00-30 were 292.71 and 123.22 $\left[ \frac{{mm}^{2}}{mmHg}\cdot{10}^{3} \right]$ for 1 mm and 2 mm, respectively.


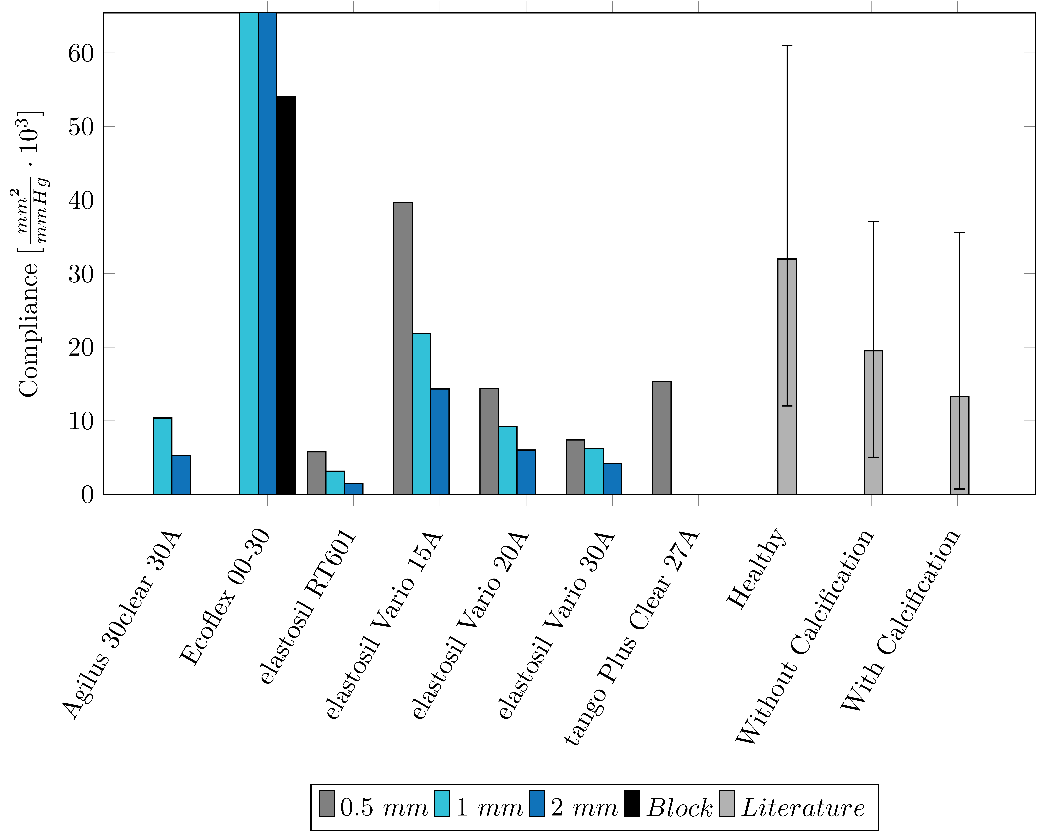


**Figure 1A.** The compliance values of tubes with an inner diameter of 3 mm and varying material and wall thicknesses were compared to the compliance values of the coronary artery from the literature [1].

## Repeatability

The repeatability of the OCT measurements was assessed by measuring one specimen four times. The results are shown in Figure 2A. The median compliance of $20.0 \left( 0.8 \right)\cdot{10}^{3}\left[ \frac{mm^{2}}{mmHg} \right]$ was calculated. The median cross-sectional area at 120 mmHg was $6.8 \left( 0.09 \right) mm^{2}$.


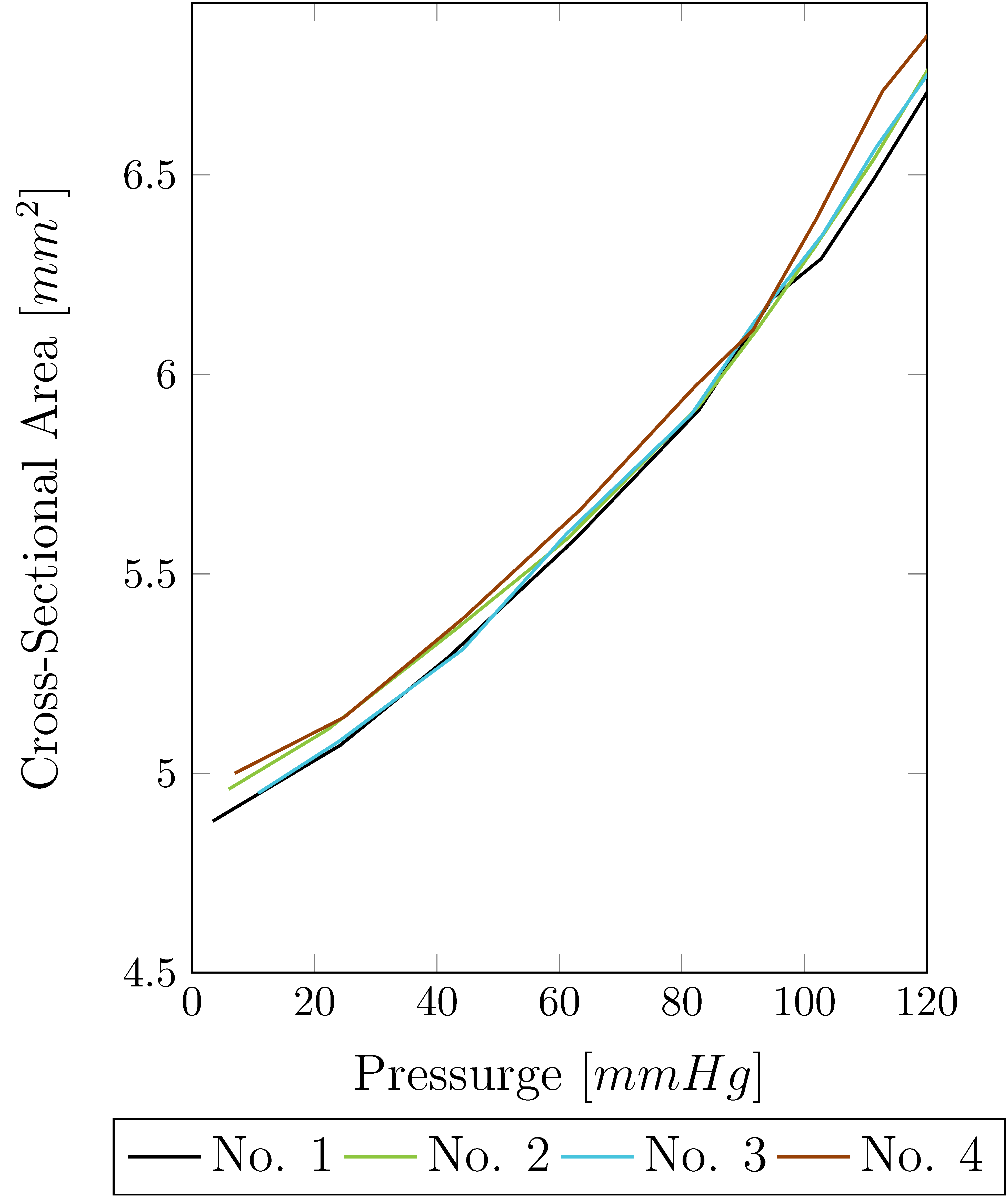


**Figure 2A.** Repeatability of the OCT measurements of one sample.

## Radial tensile properties

Radial tensile properties measured for each specimen

Table 2A: Results of the radial tensile tests for all specimen types. WT: Wall thickness, ID: Inner diameter.

| Material | n | Range | $E_{C1} \left[ kPa \right]$ | $E_{loading} \left[ kPa \right]$ | $E_{unloading} \left[ kPa \right]$ |
| --- | --- | --- | --- | --- | --- |
| WT=0.5, ID=3, Cast | 3 | 5 - 30% | 78.1 (17.2) |  |  |
|  |  | 30 - 50% | 123.4 (24) | 127.3 (21.1) | 40.9 (7.7) |
|  |  | 150 - 200% |  | 80.3 (11.1) | 78.5 (9.6) |
| WT=0.75, ID=5, Cast | 3 | 5 - 30% | 33.9 (12.2) |  |  |
|  |  | 30 - 50% | 66.1 (5.9) | 65.7 (6.5) | 33.9 (1.9) |
|  |  | 150 - 200% |  | 63.1 (3.6) | 70.9 (5) |
| WT=1, ID=3, Cast | 3 | 5 - 30% | 37.8 (4.2) |  |  |
|  |  | 30 - 50% | 64.4 (6) | 48.1 (5) | 28.1 (3.1) |
|  |  | 150 - 200% |  | 48.7 (5.5) | 53.7 (7.1) |
| WT=1, ID=5, Cast | 6 | 5 - 30% | 41.3 (3.5) |  |  |
|  |  | 30 - 50% | 107.8 (7.2) | 103.6 (6.9) | 60.1 (2.9) |
|  |  | 150 - 200% |  | 125.1 (19.8) | 159 (34.2) |
| WT=2, ID=3, Cast | 3 | 5 - 30% | 20.9 (2.3) |  |  |
|  |  | 30 - 50% | 24.7 (2.7) | 23.5 (2.4) | 16 (1.8) |
|  |  | 150 - 200% |  | 25.8 (2.8) | 24.2 (3) |
| WT=0.5, ID=3, Brushed | 3 | 5 - 30% | 45.5 (12.2) |  |  |
|  |  | 30 - 50% | 76.4 (27.6) | 62 (19.4) | 35.5 (6) |
|  |  | 150 - 200% |  | 75.8 (4.8) | 88.7 (5.2) |
| WT=0.75, ID=3, Brushed | 3 | 5 - 30% | 43.2 (3.1) |  |  |
|  |  | 30 - 50% | 81.1 (2.4) | 69.4 (2.4) | 38.2 (0.8) |
|  |  | 150 - 200% |  | 66 (1.8) | 87.7 (2.9) |
| WT=1.5, ID=3, Brushed | 3 | 5 - 30% | 53.8 (2) |  |  |
|  |  | 30 - 50% | 76.7 (3.1) | 70.7 (0.7) | 49.5 (3.6) |
|  |  | 150 - 200% |  | 68.3 (9.8) | 84.1 (10.2) |
| WT=1, ID=2.5, Stenosed w. Calcification | 3 | 5 - 30% | 50.4 (13.7) |  |  |
|  |  | 30 - 50% | 55.4 (11.8) | 49.5 (14.3) | 36.9 (11) |
|  |  | 150 - 200% |  | 62.5 (26.6) | 104.4 (50.7) |
| WT=1, ID=2.5, Stenosed w.o. Calcification | 3 | 5 - 30% | 44.5 (6.8) |  |  |
|  |  | 30 - 50% | 51.8 (5.6) | 50.4 (7.3) | 35.4 (5.3) |
|  |  | 150 - 200% |  | 53.9 (6.3) | 59.5 (10.6) |
| WT=1.5, ID=2.5, Stenosed w. Calcification | 3 | 5 - 30% | 85 (17.9) |  |  |
|  |  | 30 - 50% | 87.8 (18.7) | 83.9 (18.5) | 40.1 (3.6) |
|  |  | 150 - 200% |  | 75.9 (6.3) | 93.1 (8.5) |
| WT=1.5, ID=2.5, Stenosed w.o. Calcification | 3 | 5 - 30% | 50.1 (2.4) |  |  |
|  |  | 30 - 50% | 64.5 (4.7) | 59.2 (4.1) | 34.2 (3.3) |
|  |  | 150 - 200% |  | 62.8 (5.2) | 69.5 (8.6) |
| WT=1.5, ID=2.5, Stenosed w. Calcification | 3 | 5 - 30% | 66.3 (23.3) |  |  |
|  |  | 30 - 50% | 87.9 (30.2) | 62.4 (24) | 26.5 (12.2) |
|  |  | 150 - 200% |  | 54.4 (12.8) | 54 (22.4) |
| WT=1.5, ID=2.5, Stenosed w.o. Calcification | 3 | 5 - 30% | 69.1 (6.5) |  |  |
|  |  | 30 - 50% | 75.4 (9.3) | 71 (10.8) | 36.7 (5.5) |
|  |  | 150 - 200% |  | 60.6 (17.8) | 62.8 (27.9) |

## Measurement of the friction between PA12 and the coatings

The setup is shown in Figure 3A. The silicone tubes were bonded to a wheel (R_1_) with a diameter of 22 mm, resulting in $\beta=133.19^{\circ}$. The measurements were performed at 23°C on a Shimadzu AGS-X 10 kN (Shimadzu Corporation, Kyoto, Japan). A PA12 tube with an outer diameter of 0.8 mm and an inner diameter of 0.7 was preloaded with a weight of $F_{0}=1.0672 N$. A steel wire of 0.16 mm was inserted into the PA12 tube to avoid the deformation caused by the load. The tube was pulled through the fixed silicone specimen for 10 mm to measure the sliding friction. The resulting force $F_{fix}$ was recorded using a 200 N load cell (accuracy ± 0.5%). Furthermore, the base load of the system was measured by unlocking the wheel with the silicone specimen and pulling the PA12 tube through.


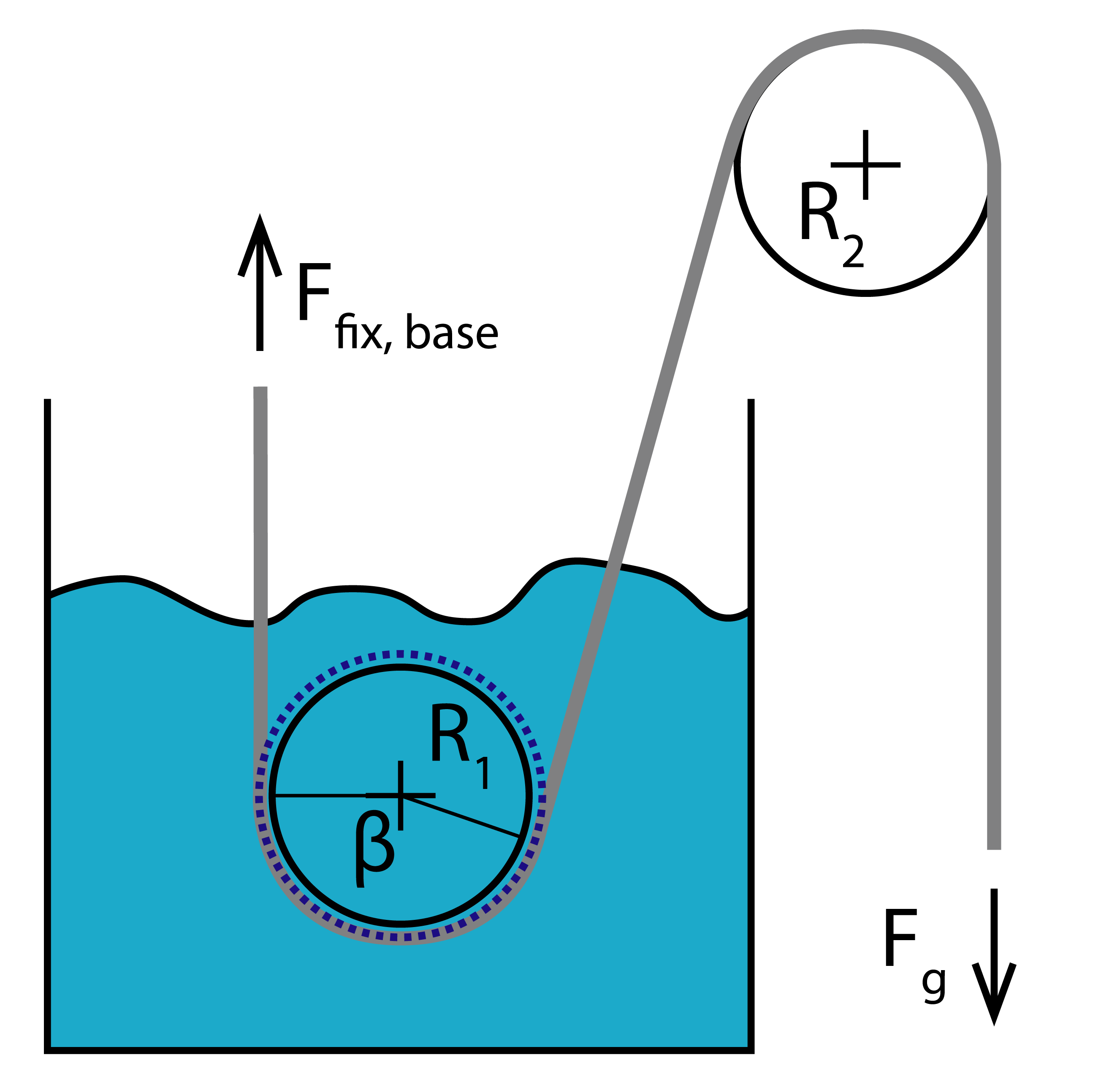


**Figure 3A.** Setup for measuring friction. After [2].

The friction was calculated using Eq. 1A [2].

| $\mu=\frac{ln(1+\frac{\bar{F_{fix}-F_{base}}}{F_{0}})}{\beta} \left[ - \right].$ | (1A) |
| --- | --- |

Notably, a small error is generated based on the internal friction of the wheel used. However, the error is the same for each measurement; therefore, the values are comparable.

**Glas beads:**

After polishing, the 3D-printed molds were placed inside a container with glass beads. Polishing causes a sticky core surface, making it easier for the beads to stick to the surface. After the surface was dry, the brushing method was used. Finally, the core was dissolved in acetone. The glass beads remained embedded into the surface of the silicone.

**Parylene C:**

The Parylene C coating was applied at Coat-X SA, La Chaux-de-Fonds, Switzerland. Two different layer thicknesses were investigated since the surface roughness of the silicone was unknown.

**PLL-g-PEG:**

The PLL-g-PEG mixture was manufactured according to [3] and was provided by Philipp Aebischer. The mixture was added to silicone tubes and left for 24 h in a +5°C refrigerator.

1. Adnan KA, Robinson C, Biggs MJ, Morgan B, Rutty GN, Borsen A, et al. P2357Measurement of coronary artery compliance and stiffness index with novel application of optical coherence tomography in re-pressurised cadaveric coronary arteries. Eur Heart J. 2017;38.

2. Blossa P, Wolfgang R, Peter W, Christian W, Alexander R, Georg Dieter K, et al. Investigations of the pushability behavior of cardiovascular angiographic catheters. Biomed Mater Eng [Internet]. 2003;13:327–43. Available from: http://iospress.metapress.com/content/CAK1U48X062DR12B

3. Aebischer P, Caversaccio M, Wimmer W. Fabrication of human anatomy-based scala tympani models with a hydrophilic coating for cochlear implant insertion experiments. Hear Res [Internet]. Elsevier B.V.; 2021;404:108205. Available from: https://doi.org/10.1016/j.heares.2021.108205
